# Pre-war experimental evidence that Putin’s propaganda elicited strong support for military invasion among Russians

**DOI:** 10.1126/sciadv.adg1199

**Published:** 2023-11-10

**Authors:** Suthan Krishnarajan, Jakob Tolstrup

**Affiliations:** Department of Political Science, Aarhus University, 8000 Aarhus C, Denmark.

## Abstract

In February 2022, Russia invaded Ukraine. The Putin regime used extensive and aggressive propaganda to win public support for the war. But can this propaganda really convince ordinary people? Using the unique timing of a survey experiment fielded a year before the invasion, we provide the first experimental evidence of the effectiveness of this propaganda among Russian citizens. Vignette treatments containing information on threats similar to stories running in Russian media around the time of the invasion in combination with statements from President Putin show that propaganda was highly effective. Even mild treatments were enough to increase support for military aggression against neighboring countries among Russians from around 8 to 48% and up to 59% among Putin’s supporters. Thus, the Russian president had good reason to believe that he could control popular opinion when he decided to launch a war against Ukraine.

## Teaser

Putin's pre-war propaganda and escalating rhetoric effectively raised support for military invasion among Russian citizens.

## INTRODUCTION

In the early hours of 24 February 2022, President Vladimir Putin addressed the Russian public in a televised speech, announcing the launch of a so-called “special military operation” in Ukraine. This marked the onset of the Russian invasion of its increasingly Western-leaning southwestern neighbor. In his speech, Putin argued that Russia was forced to make this move to defend itself against North Atlantic Treaty Organization (NATO) expansion and to protect Russian compatriots within Ukraine against what was depicted as a Nazi government in Kyiv committing genocide on Russian speakers in the breakaway republics of Donetsk and Lugansk (*1*). Putin’s half-hour address represented the first attempt at justifying war to the Russian public after the invasion had started.

In the following weeks, state-controlled media outlets pushed news stories tailored to undergird Putin’s justifications for using military force and to ease public concerns regarding the campaign. For one, the imminent threat to Russian national security was emphasized: Unsupported claims of Ukraine allegedly developing biological (*2*, *3*) and nuclear weapons (*4*–*6*) and planning a NATO-supported invasion of Donetsk, Lugansk, and Crimea (*7*, *8*) made headlines. In addition, national and local television (TV) outlets, newspapers, and radio depicted the Ukrainian government as the aggressor, claiming it was engaged in ethnic discrimination and cleansing (*9*, *10*), viciously shelling Russian-speaking residential areas in eastern and southern Ukraine (*11*, *12*), and using civilians as “human shields” to protect against the Russian forces sent to “liberate” Russia-loving Ukrainians (*13*). Journalists and editors who did not toe the official propaganda line were threatened with up to 15 years in prison, which effectively forced the closing of the last independent media outlets in the country (*14*). Although the war in Ukraine has proven more costly for Putin and his regime than he seemingly first expected, an important question remains unanswered: To what extent did regime propaganda initially make a difference in convincing ordinary Russians to support the use of military force against a neighboring country?

To examine this question, we exploit the unique timing of a preregistered survey experiment conducted in Russia on a representative sample of around 4100 respondents in February 2021; that is, 9 months before the onset of the Russian military build-up at the Ukrainian border and a year before the 2022 invasion. This timing makes it possible to assess how Russian citizens reacted to propaganda about purported hostile actions of neighboring countries in a setting that was not yet saturated by the intense state-sponsored propaganda and Putin’s many pro-war statements in the period leading up to and during the war. Nor had Russia yet reached that stage of pervasive, high-intensity repression that is likely to influence the willingness of respondents to express antiregime and antiwar preferences (*15*, *16*).

Following our preregistration plan, the experiment randomly exposes respondents to a fictional event where decision-makers in a neighboring country (Georgia or Latvia) insult and provoke Russia, either through what we term a security threat (calling Russians “weak” and announcing preparations for deployments of long-range missiles) or a cultural threat (referring to Russians as “uncultured” and announcing restrictions on the ability of the children of Russian speakers to learn the Russian language in primary schools). Although the events are fictional, the descriptions of the treatments simulate descriptions in propaganda stories typically seen in Russian news and are very similar to the kind of threat information made available by the Russian regime–controlled media before and during the invasion of Ukraine. The provocation events are followed by reactions from President Putin (no reaction, a de-escalating statement, or an escalating statement). After this, respondents declare how much they disapprove or approve of using Russian military forces against the neighboring country. The experimental design thus allows us to examine how responsive Russians were to propaganda on alleged provocations from neighboring countries and to elite cues from their president in the period leading up to the invasion of Ukraine.

As hypothesized in our preregistration plan, our results show that the Russian population’s preferences for using military force were highly manipulable. Even a relatively weak, one-time propaganda treatment—particularly in the form of a vaguely described national security threat from a militarily inferior neighboring country—was sufficient to significantly increase public support for war. After reading just one description of such an event, support for using military force against the neighboring country increased from an estimated 8 to 40% among the Russian population. If President Putin adds to this by delivering even a mildly formulated escalating statement, the number increases to 48% overall and to 59% among Putin supporters—with only 15% of Putin’s followers remaining opposed to war.

The findings provide the first-ever experimental evidence that the Russian regime could fairly easily drum up substantial initial support for military aggression and that Putin’s supporters, in particular, would follow his lead. On the one hand, our results indicate that, at least in the initial phase of the war, the Putin regime had reason to believe that it was unlikely to face substantial public pressure to stop the military operation, given that it was capable of manipulating public sentiment with relative ease and that only one of four respondents, and only 15% of Putin supporters opposed the use of military force when fed stories about security threats from abroad. This might very well have factored into Putin’s risky decision to launch a full-scale invasion of Ukraine. Once the war started, opposing opinions from ordinary Russians were aired publicly, and popular protests did take place within the first month. However, the regime quickly quashed these antiwar expressions through brute repression and even stricter censorship, and, at least in the initial months, Putin seemingly managed to maintain both high approval ratings (*16*) and high levels of support for the war (*15*).

Our results also have several broader implications for research on public opinion on war, which, until very recently, has predominately focused on democratic polities more generally and the United States in particular (*17*–*19*). We show that ordinary people in authoritarian Russia are “prudent” (*20*, *21*) in the sense that they discriminate between the severity of different types of threats and do not appear particularly belligerent in the absence of threats. These findings resonate well with recent experimental research on citizens in the communist one-party state of China (*22*, *23*) but contrast findings from survey-based research, which argues that Russians score higher on measures of so-called “blind and militant patriotism” in comparison to populations in other countries (*24*).

However, we also demonstrate that the context in which authoritarian and democratic publics form their opinions about using military force makes a crucial difference. When citizens are deprived of alternative information, critical perspectives, and different views of events—as is the case in Russia (*25*–*27*)—they become less attuned to critically assessing the information they receive (*28*–*31*). This likely makes them more susceptible to propaganda on external provocations. Our experiment even shows that respondents on the aggregate disregard important, albeit nonemphasized, differences between target countries, such as democracy levels (*22*, *32*, *33*), alliance memberships (*34*, *35*), and other relevant internal characteristics. Being treated with events in the democratic NATO and European Union (EU) member state of Latvia or the nonaligned hybrid regime of Georgia makes no difference for ordinary Russians. In our preregistration plan, we did intend to explore this issue but did not a priori formulate explicit hypotheses.

More worryingly, our results provide a potential additional explanation for the fact that personalist dictators who enjoy full control over information are indeed potentially hazardous players on the international scene [cf., (*36*, *37*)]. When relatively popular, personalist rulers such as Putin feed their people stories fabricated to trigger fear, anger, and humiliation (*38*–*40*) and they exploit their unopposed status as the leader and protector of the nation to further reinforce such feelings, they can effectively deceive ordinary citizens into thinking that military aggression is both necessary and just (*41*–*43*). The substantial leader-cue effects we find in our experiment contrast the mixed support for the elite-driven opinion formation model found in public opinion research on foreign policy in democracies [for an overview, see (*18*)]. On the basis of our experiment alone, we cannot say whether the Putin effects we find are unique or representative of a broader group of personalist authoritarian rulers, but a plausible interpretation of our results is that relatively popular authoritarian rulers of highly personalized regimes have substantially more leeway to shape public opinion on the use of military force abroad. Future research could help settle this issue by systematically investigating the issue of leader-cue effect sizes across different leaders and regimes.

However, we also show that even these broadly popular personalist autocrats such as Putin are not omnipotent. In line with the party-bias models found in democratic contexts [e.g., (*19*, *44*)] and alluded to in our preregistration plan, Putin can primarily drum up support for war among his supporters in the electorate (*44*, *45*). Thus, if the popularity of the authoritarian ruler declines [cf., (*46*, *47*)], then leader effects are likely to be reduced accordingly.

Last, and perhaps most disturbingly, and in contrast to our preregistered hypothesis, we show that Putin has a much harder time de-escalating events. Even among his own supporters, de-escalating statements by the president do not significantly reduce support for war. This contrasts with recent research on the regime in China, which shows that adjustments in media framing can move people toward more hawkish and dovish positions on Chinese foreign policy issues (*28*). Our experiment cannot show whether Putin is truly unable to de-escalate events or whether it just takes more effort than the simple de-escalating statement we use in our experiment, but it might, among other reasons, help explain why the Putin regime remained committed to continuing the war even as costs were mounting and one humiliating defeat followed another.

## RESULTS

We examine Russian citizens’ support for military aggression in a preregistered survey experiment, administered through YouGov, on a representative sample in terms of gender, age, and geography (see the “Assessing the representativeness of the survey sample” section in the Supplementary Materials for tests of representativeness) of 4144 respondents in Russia during the period of 12 February to 11 March 2021. The experiment randomly exposes respondents to a fictional event presented in a short vignette from one of the following four categories. (i) A control condition without any threats, which presents a non-threatening, albeit slightly insulting, action on the part of the government of either Latvia or Georgia: a decision to change the coloring of the country’s passport to make it look less like a Russian one. (ii) A threatening provocation from either Latvia or Georgia, which presents either a military threat or a culturally based threat: The neighboring country’s leadership has made it illegal for Russian children to learn Russian in primary schools and refers to Russians as “uncultured” (cultural threat) or the neighboring country makes funding available for the deployment of long-range ballistic missiles close to Russia’s border and refers to Russians as weak (security threat). (iii) A threatening provocation from Latvia/Georgia followed by a de-escalating statement by President Putin (“I am not greatly concerned with the developments…these actions do not constitute a direct threat”). (iv) A threatening provocation from Latvia/Georgia followed by an escalating statement by Putin (“I look with great concern to the developments… these actions constitute a direct threat”). The Methods section below presents the experimental setup in more detail.

**Fig. 1.**
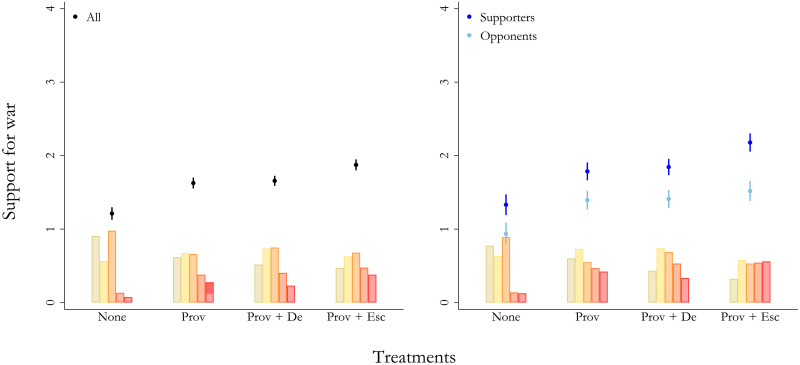
Provocations and Putin’s escalations increase support for military aggression among Russians. The *x* axis presents the control condition and the three treatment conditions. The *y* axis denotes the estimated values of support for war (0 = oppose strongly, 1 = oppose somewhat, 2 = neither favor nor oppose, 3 = favor somewhat, and 4 = favor strongly) with 95% CIs. The left panel presents the main results of the full sample given by model 1. The right panel illustrates the main results across Putin supporters (dark blue) and opponents (light blue) given by model 2. The bars at the bottom of each graph illustrate the distribution in respondents’ answers on support for war across each treatment condition (green = oppose strongly, yellow = oppose somewhat, orange = neither favor nor oppose, dark orange = favor somewhat, and red = favor strongly). In the right panel, the bars only show distributions among Putin supporters.

**Fig. 2.**
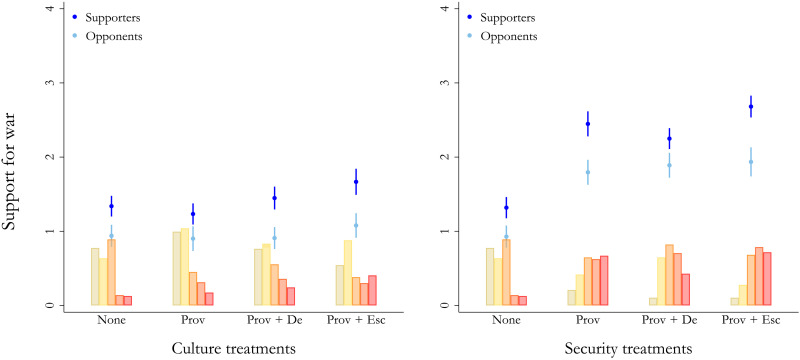
Support for military aggression among Russians is mainly driven by security provocations. The *x* axis presents the control condition and the three treatment conditions. The *y* axis denotes the estimated values of support for war (0 = oppose strongly, 1 = oppose somewhat, 2 = neither favor nor oppose, 3 = favor somewhat, and 4 = favor strongly) with 95% CIs. Results are presented across supporters (dark blue) and opponents (light blue) of President Putin given by model 2. The left panel presents results for culture treatments only. The right panel presents results for security treatments only. The bars at the bottom of each graph illustrate the distribution in respondents’ answers on support for war across each treatment condition (green = oppose strongly, yellow = oppose somewhat, orange = neither favor nor oppose, and dark orange = favor somewhat, and red = favor strongly) among Putin supporters.

The specifications of the following results are done in accordance with the preregistration plan (see the “Preregistration” section in the Supplementary Materials). For all analyses that the preregistration does not directly address—for example, results on Putin supporters versus Putin opponents (presented in Figs. 1 (right) to 4, security threat versus cultural threat (Fig. 2), and Georgia versus Latvia (Fig. 4), labeled as “exploratory” in the preregistration—the specifications follow the same logic as in the preregistered main analyses.

**Fig. 3.**
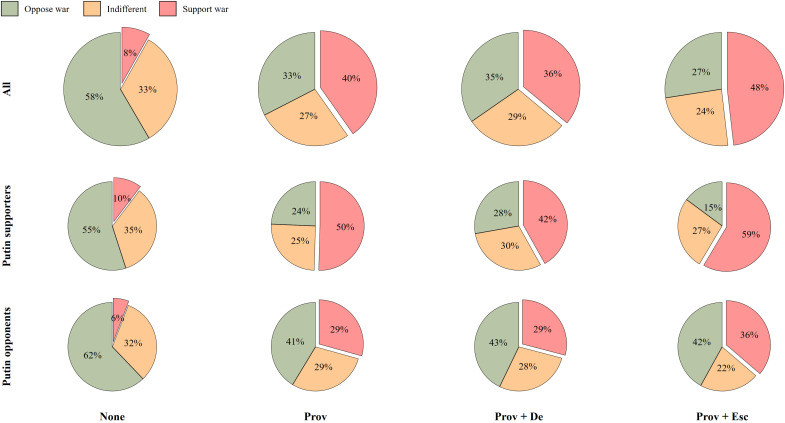
Security provocations and Putin’s escalation increase support for military aggression substantially. Charts of the estimated distributions of support for war across security treatment conditions. Green equals opposition to war (oppose strongly or oppose somewhat), orange is undecided/indifferent (neither favor nor oppose), and red equals support for war (favor somewhat or favor strongly). The top row shows distributions across all respondents, the middle row across Putin supporters, and the bottom row across Putin opponents.

Figure 1 presents the main findings on Russian citizens’ support for war across each experimental condition. The left panel presents the main results of the full sample, and the right panel provides split-sample results across opponents and supporters of President Putin based on approval/disapproval ratings among respondents measured before the introduction of the vignettes (see the “Distribution of Putin approval” section in the Supplementary Materials). Figure 2 presents split-sample results of Putin opponents and supporters across the culture and security provocations separately. In Fig. 3, we focus on the security provocations only and present estimated percentages of three collapsed categories: Green equals opposition to war (oppose strongly or oppose somewhat); orange is undecided/indifferent (neither favor nor oppose); and red equals support for war (favor somewhat or favor strongly).

### No provocation

Figures 1 and 2 show that Russian citizens generally do not support the use of military force against Georgia or Latvia. After reading the control vignette (denoted as “None” in Figs. 1 to 3), only about 8% [95% confidence interval (CI) = 5.6, 10.8] support military force against the neighboring country, as shown in Fig. 3. Although Putin supporters are significantly more supportive of war in this baseline scenario, Fig. 3 shows that the actual differences in terms of estimated percentages are quite small. In short, before the invasion and in the absence of propaganda stories tailored to invoke fear, anger, and humiliation, Russians were not a priori eager for military aggression.

### Provocation

Figure 1 demonstrates that Russians update their preferences for military action when reading about a provocation from a neighboring country (denoted as “Prov” in Figs. 1 to 3). Support for war increases by around 0.4 (95% CI = 0.30, 0.53) on the 0 to 4 scale. The distributional bars in the bottom part of Fig. 1 show that this change is driven by an actual increase in support for military aggression, not simply respondents moving from opposition to indifference to war. More specifically, when using the same categories applied to Fig. 3 (support for, indifference to, and opposition to war) on this main model specification, the average estimated proportion of the full sample of Russian respondents that support war increases from 8% (95% CI = 5.7, 10.0) in the control condition to 25% (95% CI = 22.7, 27.7) in the provocation condition [and another 25% (95% CI = 22.8, 27.8) being indifferent].

The right panel in Fig. 1 illustrates that the impact of reading about a provocation from a neighboring country is broadly similar when distinguishing between opponents and supporters of the regime. Both Putin’s opponents and supporters alike increase their willingness to support war to the same extent. However, because the baseline support levels differ, Putin supporters again generally have more favorable views of the use of military force.

Figure 2 shows that security treatments drive this effect. While the cultural provocation exerts no significant impact on Russians’ willingness to use military force against a neighboring country, security treatments trigger a substantial increase among both supporters and opponents of President Putin. Seemingly, not all types of threat scenarios are equally effective for drumming up support for war among Russians. Specifically, Fig. 3—security provocations only—demonstrates that reading about a security provocation leaves an estimated 40% (95% CI = 35.6, 44.9) of the population supportive of using military force and another 27% (95% CI = 23.0, 31.4) indifferent to it. Among Putin supporters, those numbers are even higher: Around 50% (95% CI = 43.8, 57.1) now support the use of military force, and another 25% (95% CI = 19.5, 31.0) are indifferent. In the Supplemtary materials (see the “Scrutinizing the effect of culture treatments” section in the Supplementary Materials), we present the results for the cultural treatment. Only 13% (all respondents) (95% CI = 10.2, 16.7) and 16% (Putin supporters) (95% CI = 11.8, 20.9) support using Russian military force when exposed to restrictions on the status of the Russian language in the neighboring country, while a clear majority of 71% (all respondents) (95% CI = 66.7, 75.3) and 68% (Putin supporters) (95% CI = 62.8, 74.2) oppose it. Evidently, security-related provocations make Russians substantially more willing to support military aggression, while cultural provocations alone have much less impact.

### Provocation and de-escalation

Once respondents have read about a provocation from a neighboring country, we find, contrary to our preregistered expectations, that de-escalating the situation is difficult for the Russian leader. The left panel in Fig. 1 shows that Russian citizens’ support for war is unaffected by a de-escalating statement by President Putin (denoted as “Prov + De” in Figs. 1 to 3). Somewhat unexpectedly, the right panel reveals that this is the case for Putin’s opponents and supporters alike. Only for the security treatments in the right panel of Fig. 2 do we see an effect among Putin supporters; but even here, the impact of Putin stating that he does not perceive the events in the neighboring country as a serious threat is quite small. This is also the case for the cultural treatments in the left panel of Fig. 2, where the results are not statistically significant either. In short, most Putin supporters remain, at best, unaffected by Putin’s attempts to de-escalate.

### Provocation and escalation

When Russian respondents read about a provocation from a neighboring country followed by an escalation by President Putin (denoted as “Prov + Esc” in Figs. 1 to 3), they, as expected, support military aggression even more markedly. In the left panel of Fig. 1, the average support levels increase by around 0.66 (95% CI = 66.7, 75.3) on the 0 to 4 support for war scale compared to the control condition across all respondents and both types of provocations. The average estimated proportion of Russian respondents supporting war (favor somewhat or favor strongly) on this main model specification increases up to 32% (95% CI = 29.8, 35.2) in the provocation condition [and another 26% (95% CI = 23.3, 28.3) being indifferent].

The right panel of Fig. 1 distinguishes between opponents and supporters of President Putin and shows interesting variations. Among Putin’s opponents, support for war does not increase markedly compared to the increase already captured by the provocation information itself. In short, regime opponents raise their support for the use of military force when reading about a provocation from a neighboring country, but they do not become even more supportive of war when Putin escalates the situation further.

For Putin supporters, the picture is very different. In this group, respondents even further increase their support for war. The estimated effect is around the same size as the provocation itself, ultimately increasing their support by 0.85 (95% CI = 0.66, 1.03) on the 0 to 4 scale compared to the control condition. The average estimated proportion of pro-Putin Russians supporting war (favor somewhat or favor strongly) rises to 44% (95% CI = 38.9, 48.2) in the provocation condition [and another 21% (95% CI = 17.1, 24.8) being indifferent].

Focusing only on security provocations in the right panel of Fig. 2, we see even starker increases. Reading about a security provocation from a neighboring country followed by an escalation from Putin increases support for war by 1.36 (95% CI = 1.16, 1.57) on the 0 to 4 scale compared to the control condition among Putin supporters. In contrast, cultural provocations (left panel of Fig. 2) show much weaker and nonsignificant effects. In our preregistration plan, we did not formulate explicit hypotheses on the differences between these two types of provocations.

Figure 3 further shows that after having read about a security provocation from a neighboring country followed by an escalation by Putin, an estimated 48% (95% CI = 43.4, 53.0) of all respondents and a whopping 59% (95% CI = 52.0, 65.1) of Putin supporters favor war against the neighboring country—leaving only an estimated 27% (95% CI = 23.2, 31.8) among the entire population and 15% (95% CI = 10.1, 19.6) of Putin supporters disapproving of military action. In other words, around half of the Russian population and close to two-thirds of Putin’s supporters are estimated to be in favor of war against a neighboring country after having read about a security provocation from that country followed by an escalation by Putin. For the cultural treatment, the effects are much weaker, as an estimated 22% (95% CI = 17.7, 25.7) of all respondents and 28% (95% CI = 22.1, 34.1) of Putin supporters hold preferences in favor of using military force against a neighboring country following Putin’s escalating remark, and 63% (all respondents) (95% CI = 60.0, 67.3) and 57% (Putin supporters) (95% CI = 50.0, 63.3) remain opposed to military aggression. Still, it is noteworthy that even such a low-threat scenario produces an about 18 percentage point increase in support for war (compared to the control situation) among those Russians who support Putin.

### Adversary country

**Fig. 4.**
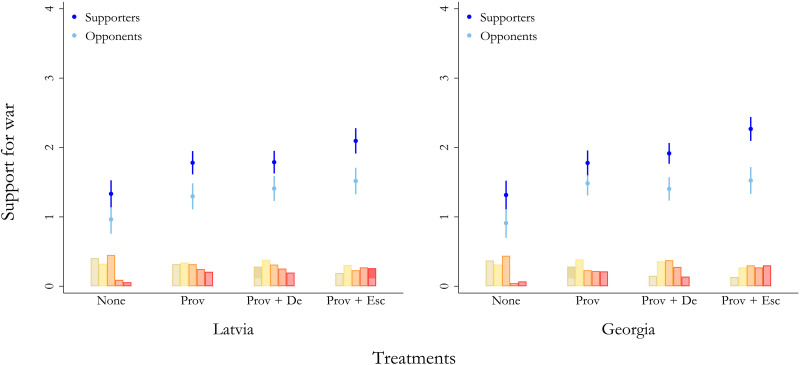
Effects are similar across Latvia and Georgia. The *x* axis presents the control condition and the three treatment conditions. The *y* axis denotes the estimated values of support for war (0 = oppose strongly, 1 = oppose somewhat, 2 = neither favor nor oppose, 3 = favor somewhat, and 4 = favor strongly) with 95% CIs. The left panel presents the results for Latvia only, while the right panel illustrates results for Georgia across Putin supporters (dark blue) and opponents (light blue) given by model 2. The bars at the bottom of each graph illustrate the distribution in respondents’ answers on support for war across each treatment condition (green = oppose strongly, yellow = oppose somewhat, orange = neither favor nor oppose, dark orange = favor somewhat, and red = favor strongly).

The experiment varies the adversary country, as respondents receive information on either Georgia or Latvia to analyze whether factors such as NATO and EU membership, democracy level, and the size of the country’s Russian-speaking minority affect the findings. As Fig. 4 shows, we find no significant differences between the treatments involving Latvia and those concerning Georgia.

### Benchmarking the effects

How strong are these effects? Table 1 summarizes some of the most well-known studies on public support for war. Effect sizes in previous studies vary markedly, from around five percentage points [e.g., (*48*)] up to approximately 33 percentage points [e.g., (*34*)]. Yet, most effect sizes are around 7 to 14 percentage points on support for war (*22*, *32*, *49*). From this vantage point, the results from our study demonstrate a comparatively large effect of a one-time vignette treatment. In particular, the substantial effects of statements from Putin on his supporters’ willingness to use military force contrasts with the inconsistent elite cue effects found by previous studies [e.g., (*18*)]. On top of this, given that we know respondents are more likely to support war in surveys with fictitious country names (*50*), the substantial effects we find in a survey with real countries further attest to the strength of the results. It suggests that the findings of our study, both with respect to provocations and leader cues, are not only consistent but also substantial.

## DISCUSSION

Most research on public support for war has focused on democratic regimes more generally and the United States in particular (*17*–*19*). Hence, our understanding of the circumstances under which dictators can foster popular support for war remains poor. This empirical gap in existing research is unfortunate for two reasons. First, authoritarian regimes, particularly personalist dictatorships such as the one found in Russia, are, on average, more belligerent than democracies (*37*, *43*). As the authoritarian form of government (*54*), and the personalist form in particular (*55*, *56*), has gained ground worldwide during the last two decades, it is crucial to study these regimes if we want to better understand war and peace globally. Second, as mass-based uprisings against dictatorships have become more widespread (*57*), and autocrats increasingly rely on some sort of electoral legitimacy (*58*, *59*), authoritarian leaders are becoming more sensitive and attentive to popular support (*60*, *61*). Insights into the interplay between the ruler and the people are thus essential for understanding contemporary authoritarian conflict patterns.

From research on democracies, we know that securing popular support is crucial for modern warfare (*62*). Antiwar mobilization and broader public disillusion with ongoing military campaigns exacerbate the perceived economic and human costs of waging war [pp. 38–39 of (*63*), (*64*)], negatively affect leader approval ratings [pp. 1932–1936 of (*17*), (*65*, *66*, *69*)], and more generally weaken the incumbent’s political maneuverability [pp. 850 of (*32*)]. As the Russian invasion of Ukraine and recent research (*42*, *67*) illustrate, autocrats also pay careful attention to and vigorously try to shape public opinion to avoid the pitfalls associated with public antiwar sentiments.

Our analysis of a Russian survey experiment meaningfully improves our knowledge of this understudied topic. It casts new light on the ongoing Russian war in Ukraine, which has been described as the end of the post-Cold War order (*68*). Six key findings deserve further discussion.

First, as hypothesized in our preregistration, we show that ordinary citizens in an autocracy such as Russia are highly sensitive to the threat information they receive when forming preferences on the use of military force. As the differences between our control and treatment groups show, Russians correctly evaluate degrees of threat and discriminate between different threat types, clearly responding more strongly to security threats than what we term cultural threats. In this sense, the Russian public reacts “predictably” and “prudently,” as public opinion research in democracies has also found (*20*, *21*). Moreover, and quite reassuringly, we show that ordinary people in Russia are not particularly belligerent at the outset. Only 8% of the respondents in our experiment support military use of force against a neighboring country that does not engage in threatening actions. These findings resonate with recent experimental research on authoritarian China, showing that the Chinese are not all that different from citizens in democratic Western states (*22*, *23*). Research that is based on ordinary surveys has highlighted how Russian citizens are more committed to so-called “blind and militant patriotism” (*24*), at least in comparison to new and old democracies in the Western world. Yet, our experiment shows that strong baseline preferences for this malign form of patriotism do not necessarily in and of themselves translate into high baseline support levels for military aggression.

However, our study also highlights a second point: Prudence has its limits. Deprived of both supplementary information concerning the neighboring countries in question and alternative perspectives on the credibility of the events on the ground and their implications, many Russians are fairly easily convinced that a military response is indeed necessary.

As the results demonstrate, we found no significant differences between the treatments involving Georgia and those concerning Latvia. That is, respondents did not “properly” weigh costs and benefits across the two target countries (see also the “Main results for Latvia and Georgia separately” section in the Supplementary Materials). Although we did not preregister these expectations, we should, for example, expect a military threat from Georgia—a country Russia has recently, in 2008, waged war against—to be more credible and less costly than attacking Latvia—a NATO and EU member state (*35*). Likewise, the impact of language restrictions in Georgia would be much smaller because the Russian-speaking minority now makes up less than 1% of the population. For Latvia, the number is around 25%. Last, according to the democratic peace theory (*32*, *33*)—for which a recent study also found support in an authoritarian setting (*22*)—we should expect Russians to be less willing to support strikes against the consolidated democracy in Latvia in comparison to Georgia’s hybrid regime. Thus, for respondents treated with the security threat, we should expect more willingness to support military aggression against Georgia relative to Latvia. Among those treated with the cultural threat scenario, expectations are less clear-cut. In Latvia, the threat of curtailing the rights of Russian speakers is more substantial, but the potential costs of military action are also higher for Russia. Nonetheless, none of these country differences appears to matter for Russian citizens’ willingness to support a war, at least not as long as they are not accentuated.

In a regime with tight information control such as Russia (*25*), respondents are likely to be deprived of this essential contextual information, severely constraining their ability to act as prudent citizens [cf., (*29*, *30*, *69*)]. In the Reporters Without Borders’ Press Freedom Ranking, Russia was placed 150 of 180 countries in 2021 and moved down to number 155 in 2022 (*70*). According to Levada polls (*71*), state-controlled TV remains the main source of information for around 65% of Russians, and more than 50% watch it every day or almost every day. Even among Russians aged 18 to 24, 44% use TV as their main source of news information. The situation is not necessarily any better for those people who get most of their information from social media and internet news sites. The regime controls all the most popular newspapers, and their online content is heavily biased. Even the most widely used news aggregator, *Yandex.News*, and the search engine Yandex are constructed to promote regime-controlled sources and demote independent ones (*25*). Thus, if the public debate surrounding military campaigns does not attend to, for example, potential costs and risks, ordinary people are forced to independently seek out information that is vital for assessing the pros and cons of engaging in war. Citizens in propaganda-penetrating societies might therefore very well act more in accordance with what has been termed the “Almond-Lippmann consensus” (*72*): That public opinion on foreign policy is poorly informed and unstructured.

Third, public opinion research on foreign policy in democracies has found mixed support for the elite-driven opinion formation model [for an overview, see (*18*)]. However, the leader-cue effects we expected in our preregistration and found in our analysis are substantial. Respondents with a positive view of President Putin markedly increase their support for war when Putin frames events as “threatening” and necessary to “counter.” In the real-world events that unfolded in early 2022, Russians were exposed to lengthy speeches and numerous statements by Putin, all emphasizing the severity of threats to Russia and the necessity of a military response. Although counterarguments from, say, opposition figures or leading civil society actors would likely drive down the treatment effects we observe in our experiment, these alternative voices were simply not available to the large majority of Russians around the time of the invasion. And even for those who did encounter antiwar views online, these alternative voices were likely to be drowned out in the flood of regime-controlled or regime-influenced content. Tellingly, our results show that the effects are consistent across different media habits, regardless of whether citizens get their daily news from television, the internet, or social media (see the “Examining the impact of media usage” section in the Supplementary Materials).

Fourth, research conducted in democratic countries has studied whether and how party bias affects leader-cue effects in foreign policy opinion formation [for an overview, see (*18*, *19*, *44*)]. As alluded to, although not explicitly hypothesized, in our preregistration plan, our analysis shows that acceptance of elite cues in Russia is generally highly conditional on initial support for Putin. While supporters of Putin are ready to follow his lead, those who disapprove of him are much more hesitant. This means that, as long as the Russian leader enjoys widespread popularity (*47*, *73*)—which recent research shows he has, at least up to mid-2022 (*16*)—he is well positioned to shape public opinion, including on decisions concerning war and peace.

Unfortunately, with our experimental setup, we cannot assess whether the elite-cue effect we observe can truly be attributed to Putin personally as we did not vary the sender of the escalating statements. Again, while statements from nonregime elites or perhaps even Western leaders might have generated different results, we find it unlikely that substituting statements from Putin with that of other top officials within the Russian regime would affect our findings. The reason is not that we think that Russians would be equally likely to follow, say Foreign Minister Sergey Lavrov or Defense Minister Sergey Shoigu, but rather that in the lead-up to the war in Ukraine, Putin impersonalized the regime in Russia, and most of his closest associates did not express independent policy views. And for this reason, it is very likely that Putin supporters simply automatically think of Putin when Shoigu, Lavrov, or other members of the inner circle make their statements. Isolating the “true Putin effect” is thus a tricky issue in the Russian context.

Fifth, in contrast to our preregistered expectations, and perhaps most alarmingly, we found an asymmetry between the effects of Putin’s escalating and de-escalating cues. While recent research from China shows how modifications in media framing can move people toward more hawkish and dovish positions on foreign policy issues (*28*), we find that even among the Russian president’s supporters, de-escalating statements do not significantly reduce support for war. It may be that Putin truly is unable to de-escalate the situation, which would speak in favor of the “blind and militant patriotism” perspective (*28*) discussed above. That is, Russians tap into aggressive patriotism as soon as they receive particular cues, and pulling them out of this automatic reasoning again is indeed quite hard—even for the main cue provider, the President. Among other reasons, this could help explain why Putin remains committed to continuing a war that is both costly and potentially damaging to his regime. However, it may also be that Putin can in fact de-escalate tension if need be but that it just takes more effort than the simple de-escalating statement used in our experiment. Last, it might be the case that some respondents read the statement not as evidence that Putin is trying to de-escalate events but rather that he is belittling the enemy when noting that “these actions do not constitute a direct threat.” For some respondents, this could perhaps signal not that Russia should not respond to the threat but rather that Russia will for sure prevail if it comes to military confrontation. Unfortunately, we cannot assess which of these different interpretations best explains these unexpected results.

Last, and more generally, our survey experiment shows that propaganda works (*28*, *31*). It is an effective tool for increasing public support for military intervention. It is even more effective when combined with escalating cues from a broadly popular ruler who is typically presented as the knowledgeable, caring, and resolute leader in all dominant media outlets (*74*, *75*). However, our survey experiment captures only the unopposed, clean propaganda effect before the actual invasion of Ukraine in February 2022. We cannot say whether the propaganda and leader-cue effects we find are robust over time and to changes in the environment Russians live in.

Once the war started, we saw opposition voices speak up and protesters in the streets opposing the war. While the propaganda apparatus continued spinning, the regime needed to step up repression and censorship efforts to quell dissent. In the short run, coercion worked. Larger protests no longer occurred, and critical voices were silenced one after another. During the first months following the invasion, Putin even seemed to have maintained broad popular support among the electorate (*16*), and support for the war also remained strong (*15*). However, as the economic and human costs of war for ordinary Russians have grown over time, so have the challenges the Putin regime faces in upholding public support for the war in Ukraine. Although reliable evidence is scarce, we do have indications that Russians by the end of 2022 had become more uncertain and seemingly less supportive of the war (*76*), and artificial intelligence analyses of social media content produced by ordinary Russians suggest that the effectiveness of regime propaganda in producing positive sentiments in the population is diminishing (*77*). Part of the explanation may be that people now seek alternative, independent information. Recent research shows that citizens in closed autocracies are more inclined to seek out alternative information during periods of severe crisis (*78*). After some time, this also happened in Russia, as indicated by the hundreds of thousands of daily virtual private network (VPN) downloads in April and May 2022 that allowed citizens to access blocked news sites and receive unbiased information (*79*). Tellingly, the Russian regime has tried to counter this trend by attempting to block access to VPNs and using misinformation campaigns to dissuade this software, warning that VPNs are unsafe and make users digitally vulnerable.

In summary, this study demonstrates that when Putin launched the war in February 2022, he had good reason to expect that he could drum up broad support for his mission as long as he controlled the information space. For Putin, war against Ukraine was not necessary, but control of public opinion made it a feasible and perhaps even attractive operation. Time will show whether Putin’s gamble proves successful or eventually accelerates his downfall.

## METHODS

We examine Russian citizens’ support for war in a preregistered survey experiment, administered through YouGov, on a representative sample in terms of gender, age, and geography of 4144 respondents in Russia during the period of 12 February to 11 March 2021.

### Ethical concerns

The experiment and data collection process have been approved and registered at the Data Protection Unit at the university of the authors. By assigning our project to this Institutional Review Board, our survey data collection was subject to an array of restrictions and guidelines monitored by the board.

In contemporary Russia, citizens face a severe risk of punishment for providing statements considered disloyal by the regime, especially regarding Russia’s war efforts in neighboring countries. Ensuring respondents’ anonymity and proper storage of sensitive survey data is therefore of vital importance. Both issues were dealt with following the rules of this board. In addition, we thoroughly discussed survey wording and sensitivity issues with YouGov and the Russian survey partners before, during, and after the data collection process. We paid particular attention to ensuring participants’ consent throughout the entire survey. Respondents could freely choose not to answer any questions or opt out of the survey at any time without losing any rewards. Last, at the end of the survey, respondents received thorough debriefing information. We formulated the debrief in a manner easy to understand by relying on everyday Russian language. We explained that the vignette information respondents received was fictitious. We explicitly stated that Russian relations with Latvia/Georgia had not deteriorated as described in the survey, and we explained that the use of these fictitious events is important for research on and our understanding of the causes of peace and conflict. In the Supplementary Materials (see the “Ethical considerations” in the Supplementary Materials), we discuss these and additional ethical issues in more detail.

### Experimental structure

The experiment randomly exposes respondents to a fictional event presented in a detailed yet concise vignette. Respondents are randomly assigned to one vignette from one of the following four categories: a control condition, a provocation from Latvia/Georgia (consisting of both an insulting remark and a threatening action), a provocation from Latvia/Georgia followed by a de-escalating statement by President Putin, or a provocation from Latvia/Georgia followed by an escalating statement by Putin.

Within these four overarching categories, the experiment randomly varies in two additional ways. First, it varies on the type of provocation. One provocation concerns Russian culture (broadly understood) by focusing on Latvia’s/Georgia’s treatment of the language rights of Russian-speaking minorities within their borders and decision-makers referring to Russians as “uncultured”; the other provocation concerns Russian security and focuses on potential missile deployment by Latvia/Georgia and decision-makers referring to Russians as weak.

Second, the experiment varies the country as respondents receive information on either Georgia or Latvia. Both Latvia and Georgia were bound together with Russia in the Soviet Union. They both share borders with Russia and are small countries with little military and economic power. This makes the two countries highly comparable as potential targets for Russian military action. On the other hand, they differ regarding potentially important factors such as NATO and EU membership, democracy level, and the size of their Russian-speaking minorities.

Randomizing these two factors within the overarching four experimental conditions allows us to account for several alternative explanations for our Russian respondents’ preferences for or against war. Table 2 presents the different experimental conditions. We set up the survey in Russian (i.e., respondents read and answered all questions in Russian), but in what follows, all text descriptions from the survey are shown in an English translation.

### Main experimental conditions

As described above, the experimental design includes four overarching conditions. The control condition presents a nonthreatening, albeit slightly insulting, action on the part of the government of the neighboring country: a decision to change the coloring of the country’s passport to make it look less like Russia’s. This trivial action serves as the reference point for measuring the impacts of the following treatment conditions.

The provocation condition presents a threatening provocation, either by noting that the neighboring country’s leadership has made it illegal for Russian children to learn Russian in primary schools and referred to Russians as “uncultured” or by noting that the neighboring country has made funding available for the deployment of long-range ballistic missiles close to Russia’s borders and referred to Russians as weak. That is, the provocations include two parts: an action (e.g., making funding available for the deployment of long-range ballistic missiles close to Russia’s borders) and an insult (e.g., referring to Russians as weak). Pilot results reveal that any effects of provocations are likely induced by the provoking action rather than the insults (see the “Results from the pilot study” in the Supplementary Materials). Still, both components are included to increase the realism of how provocations are often framed in Russian media.

The last two conditions add to the provocation by including a statement from President Putin. Under the de-escalating condition, Putin is unconcerned with the described event (“I am not greatly concerned with the developments … these actions do not constitute a direct threat”). Under the escalating condition, the event does concern the Russian president (“I look with great concern to the developments … these actions constitute a direct threat”).

### Imitating Russian propaganda

The vignettes are fictional but describe scenarios that are realistic in the sense that they touch upon salient issues. Policies toward Russian-speaking minorities in neighboring countries, generally, and the status of the Russian language, in particular, have been highly salient issues in Russian politics throughout the post-Soviet period (*80*, *81*), as have national security threats in general (*82*, *83*).

**Fig. 5.**
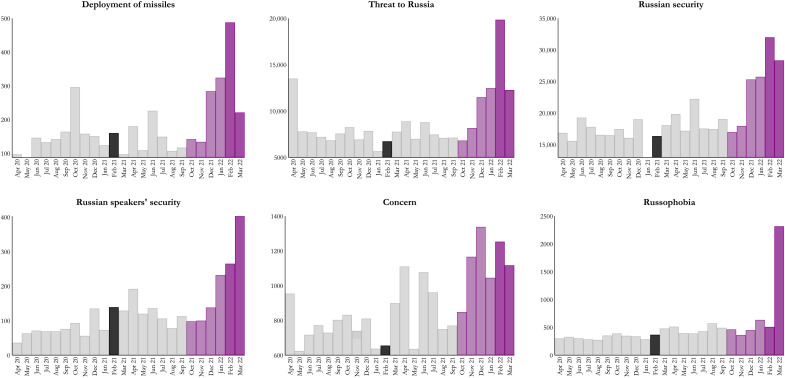
Key vignette terms became highly salient in Russian media before the invasion of Ukraine. Total number of references to “Deployment of missiles (Развертывание ракет),” “Threat to Russia (Угроза России),” “Russian security (Безопасность России),” “Russian speakers’ security (Безопасность русскоязычных),” “Concern (беспокойство),” and “Russophobia (Pусофобия)” in all Russian media included in the Factiva database (the search was performed on 6 April 2022). Black bar denotes the month of the survey. Light purple bars represent months of Russian military build-up at the Ukrainian border. Dark purple shows the months of the Russian invasion of Ukraine.

Moreover, the vignettes imitate stories and even use some of the same phrases or words that have appeared in the regime-controlled media in Russia. This is the case both for the provocations [for example, media stories featuring discrimination of ethnic Russians living in neighboring countries; see (*84*, *85*, *86*); for example, media stories describing security-related threats in neighboring countries; see (*87*, *88*, *89*); for example, stories featuring provocative statements from government officials of neighbroing countries; see (*90*, *91*–*93*)] and the statements by President Putin [see, e.g., (*94*, *95*, *96*)]. The vignette descriptions come very close to the kind of propaganda stories that Russians were, in fact, exposed to in the months leading up to and during the war in Ukraine. Stories on decision-makers in neighboring countries making insulting comments are of course less common, but we include them because the broader theme of derogation of Russians is pervasive in Russian state propaganda (typically encapsulated in the term “Russophobia,” which is used to refer to inexplicable hatred of Russia and Russians). To verify that the Russian media has run stories with threat information and language similar to what we use in the experiment, we searched the Factiva database for relevant words/phrases: “Deployment of missiles (Развертывание ракет),” “Threat to Russia (Угроза России),” “Russian security (Безопасность России),” “Russian speakers’ security (Безопасность русскоязычных),” “Concern (Беспокойство),” and “Russophobia (Pусофобия).” Figure 5 shows the distribution of the total monthly number of references in all Russian media included in the database to these particular words across a period of 2 years.

The histograms demonstrate three things. First, these words were widely used in Russian government-controlled media outlets during the past 2 years. Second, these words were much more frequently used in the Russian media leading up to and after the Russian invasion of Ukraine in late February 2022. The information Russians received in the form of government propaganda thus bears much resemblance to the vignettes they read in our study. Stories are presented as facts, and the phrases used are very similar. Last, Fig. 5 shows how the exact words figured much less frequently when we ran the survey, in February–March 2021. We are thus able to capture a cleaner propaganda effect with our data than what would have been the case if we had run the survey after the invasion when the intense regime propaganda would have heavily “pretreated” respondents and the greater reliance on brute oppression that the Putin regime quickly resorted to would likely have biased the results.

### Outcome variable

After reading the vignette, respondents answer whether they support using Russian military force against Georgia (or Latvia) to stop these developments. Answer categories are measured on a five-point 0 to 4 scale (oppose strongly, oppose somewhat, neither favor nor oppose, favor somewhat, or favor strongly).

Based on these developments, would you favor or oppose using Russian military force against Georgia (Latvia) to stop this?

This formulation is similar to the official language that was used by the upper house in Russia, the Federation Council, and transmitted by national regime-controlled media when granting the president the right to use military force in Crimea in 2014, Syria in 2015, and Ukraine in 2022 (*97*–*99*). In Russian, it thus signifies something resembling a larger-scale intervention, thus precisely tapping into the phenomenon we analyze: support for war.

Moreover, our chosen formulation is comparable to previous studies on public support for war. Table 1 demonstrates that despite variations in question wordings in existing studies, there seems to be a common denominator in that none uses the word “war” but rather inquires about the “use of [country’s] military.” Our study is similar in this regard, as it inquires respondents about “using Russian military force.”

**Table 1.** Sample of existing literature. Nonexhaustive list of studies that experimentally examine support for war among citizens. “Research question” denotes the main (or one of the main) research question(s) of the study in question. “Question wording” denotes the specific question wording of the outcome question measuring support for war. “% Support for war” provides numbers for the proportion of citizens who support war across various experimental conditions. N/A, not applicable.

Study	Research question	Question wording	% Support for war
Tomz and Weeks (*32*)	Are citizens in democracies less likely to support war against other democracies?	Would you favor or oppose using the U.S. military to attack the country’s nuclear development sites?	U.S.: 53.3% (not democracy) and 41.9% (democracy); U.K.: 34.2% (not democracy) and 20.9% (democracy)
Tomz and Weeks (*34*)	How do military alliances affect support for war in attempts to defend victims of aggression?	Do you favor or oppose sending the U.S. military to stop the invasion?	U.S.: 79% (victim alliance) and 46% (victim not alliance)
Tomz and Weeks (*51*)	Do human rights practices of foreign adversaries affect support for war?	Do you favor or oppose using the U.S. military to attack the country’s nuclear development sites?	U.S.: 57% (violates human rights) and 40% (respects human rights)
Tomz and Weeks (*49*)	Do people support military retaliation against foreign countries that have interfered in elections at home?	If the 2024 election happened just as we described, which policies would you support or oppose? Launch a military strike against country]	U.S.: 19% (foreign operational election interference) and 12% (no election interference)
Kertzer and Zeitzoff (*18*)	Do cues from social peers affect support for deployment of military forces?	N/A	Results presented as marginal effects only.
Carnegie *et al.* (*52*)	Do citizens support the use of covert military force against other democracies?	A US president deliberated with his advisors about whether to conduct a military operation against country B. [...] Given the facts described in the scenario, do you support the U.S. government’s actions?	Results presented as marginal effects only.
Bell and Quek (*22*)	Are citizens in autocracies less likely to support war against democracies?	Would you favor or oppose the Chinese military participating in an attack on the country’s nuclear development sites?	China: 38.8% (nondemocracy) and 32.9% (democracy)
Johns and Davies (*48*)	Are citizens in democracies less likely to support war against other democracies?	On a scale from 0 (strongly oppose) to 6 (strongly support), how do you feel about British/American air strikes in this case?	U.S.: 49.4% (not democracy) and 44.7% (Democracy); U.K.: 41.0% (not democracy) and 34.7% (democracy)
Masterson (*53*)	Does humiliation increase support for military conflict?	If the attacker cannot be talked into withdrawing, should our government use our military to push back the invaders, or should we stay out of it?	Results presented as marginal effects only.

**Table 2.** Treatment vignettes (Georgia example), translated into English.

	Control	Provocation	Provocation + de-escalation	Provocation + escalation
Cultural	Yesterday, Georgia’s government passed a law that changes the design of the country’s passport to make it look less similar to the Russian one.	Yesterday, Georgia’s government passed a law that makes it illegal for Russian children in Georgia to learn the Russian language in primary schools. During the following press conference, Georgia’s prime minister referred to Russians as “uncultured.”	[Provocation] + President Putin has made the following statement regarding the situation: I am not concerned with the developments in Georgia. These actions do not constitute a direct threat to our special Russian culture or the uniqueness of the Russian people.	[Provocation] + President Putin has made the following statement regarding the situation: I look with great concern to the developments in Georgia. These actions constitute a direct threat to our special Russian culture and the uniqueness of the Russian people. They must be countered.
Security		Yesterday, Georgia’s government passed a law that provides funding for the deployment of long-range ballistic missiles to an area in close proximity to the Russian border. During the following press conference, Georgia’s prime minister referred to Russians as ‘weak’.	[Provocation] + President Putin has made the following statement regarding the situation: I am not greatly concerned with the developments in Georgia. These actions do not constitute a direct threat to the security of our country or that of the Russian people.	[Provocation] + President Putin has made the following statement regarding the situation: I look with great concern to the developments in Georgia. These actions constitute a direct threat to the security of our country and the Russian people. They must be countered.

### Moderating variable

Before reading the vignette, respondents declared their approval/disapproval of President Vladimir Putin.

In general, do you approve or disapprove of the way Vladimir Putin is handling his job as president?

The answer categories are measured on a five-point 0 to 4 scale (strongly disapprove, somewhat disapprove, neither approve nor disapprove, somewhat approve, and strongly approve). In the analyses, we group respondents who approve of President Putin (somewhat approve and strongly approve) as Putin supporters and respondents who disapprove of President Putin (strongly disapprove and somewhat disapprove) as nonsupporters. In the Supplementary Materials (see the “Distribution of Putin approval” section in the Supplementary Materials), we present the distribution of Putin’s approval and we discuss and address potential issues of social desirability bias; that is, the risk that respondents will not declare their true opinions on President Putin.

### Estimation method

The main estimation strategy consists of a series of OLS models, which take the following two general forms

Model 1

$$M_{i}=\beta P_{i}+\delta D_{i}+\varphi E_{i}+\varepsilon_{i}$$

Model 2

$$M_{i}=\beta P_{i}+\delta D_{i}+\varphi E_{i}+\vartheta A_{i}+\theta (P_{i}A_{i})+\lambda (D_{i}A_{i})+\zeta (E_{i}A_{i})+\text{γ}X_{i}+{\text{ε}}_{i}$$

for *i* = 1, …, *n* as respondents.

Model 1 constitutes the main analysis with a simple model specification. It examines the average marginal effects of the three treatment conditions on support for war. The dependent variable, M_*i*_, is a given respondent’s support for using Russian military force against Georgia (or Latvia). The following three variables represent the three treatment conditions. P_*i*_ constitutes the provocation vignette, D_*i*_ is the vignette with provocation followed by a de-escalating message from President Putin, and E_*i*_ represents the vignette with a provocation followed by an escalating statement from Putin. The vignette without any real provocation (i.e., the control condition) is excluded and provides the reference point for the marginal effects of the three treatment conditions.

Model 2 examines the main analysis across supporters and opponents of President Putin, given by the dummy variable, A_*i*_, taking the value 0 for respondents who strongly disapprove or somewhat disapprove of Putin and 1 for respondents who somewhat approve or strongly approve of Putin (not including those who answer “neither approve nor disapprove” in these specific analyses). The model specification includes the product terms of each treatment condition, P_*i*_, D_*i*_, and E_*i*_, and the support for Putin variable, P_*i*_. This allows us to examine whether Putin’s supporters and opponents react differently to the various treatment conditions. **X**_*i*_ is a vector of control variables, included to account for potential confounders of support for Putin. It includes gender, age, region, and income.

In most analyses, cultural and security vignettes are collapsed, while in some (see, e.g., Fig. 2), results are shown for both types of provocations separately.

ε_*i*_ is the error term, and all models use robust SEs. Results are presented as figures and charts to ease visual interpretation (see the “Regression table of main results” section in the Supplementary Materials for all regression tables).

## Supplementary Materials

### This PDF file includes:

Supplementary Text Pre-registration information Survey questionnaire Figs. S1 to S14 Tables S1 to S3 Legend for data S1
