# Supplementary material for: Pre-war experimental evidence that Putin’s propaganda elicited strong support for military invasion among Russians
Source: Sci Adv. 2023 Nov 10;9(45):eadg1199. doi: 10.1126/sciadv.adg1199 (PMC10637735; doi:10.1126/sciadv.adg1199)
Supplement: Supplementary file 1 — Supplementary Text Pre-registration information Survey questionnaire Figs. S1 to S14 Tables S1 to S3 Legend for data S1 [file sciadv.adg1199_sm.pdf]

Science Advances
AAAS

Supplementary Materials for

Pre-war experimental evidence that Putin's propaganda elicited strong support for military invasion among Russians

Suthan Krishnarajan and Jakob Tolstrup

Corresponding author: Suthan Krishnarajan, suthan@ps.au.dk

Sci. Adv. **9**, eadg1199 (2023)
DOI: 10.1126/sciadv.adg1199

The PDF file includes:

Supplementary Text
Pre-registration information
Survey questionnaire
Figs. S1 to S14
Tables S1 to S3
Legend for data S1

Other Supplementary Material for this manuscript includes the following:

Data S1

A: Pre-registration

This section presents the pre-registration of this study. All aspects of this study were pre-registered: expectations, experimental design, survey questionnaire, data collection procedures, measurement of all key variables, model specifications, and estimation methods.

Below, a screenshot of the pre-registration page is shown (https://osf.io/bmf4w/?view_only=ce69d0e4f25e460e80a2a66a9cab83b3), followed by the text of the full pre-registration. Further below we present deviations from the pre-registration plan.

Deviations from pre-registration

Most parts of the pre-registration plan were implemented in the article. We deviate from the pre-registration with regard to four elements:

1) The article examines both the effects of provocations and the impacts of President Putin's statements. The pre-registration focuses mainly on the latter part of the experiment.

2) The article does not examine the theoretical mechanisms but focuses instead on the main effects of propaganda (provocations and statements from President Putin). This restriction is due to space constraints, but the results of the mediation analyses show strong and consistent mediation effects of almost all measured theoretical mechanisms, as expected.

3) In our preregistration plan, we did not formulate explicit hypotheses on the differences between the two types of provocations, Putin supporters and opponents, and adversary countries but referred to these as exploratory issues. In the article, we explore these differences in greater detail to cast more light on the effectiveness of different types of propaganda on different types of audiences in relation to different types of targets for military action.

4) The title of the manuscript has been changed.

Apart from this, the manuscript follows the pre-registration plan.

OSF REGISTRIES Add New My Registrations Help Donate

Follow the Leader! How Putin's Rhetoric Shapes Russian Popular Support for War against Neighboring Countries.

Updates

Overview
Files
Wiki
Components 0
Links 0
Analytics
Comments 0

Study Information

Hypotheses

Authoritarian regimes are characterized by a low degree of political pluralism and a high degree of control over information. In theory, this makes it easier for authoritarian governments to drum up public support for the use of military force. We therefore expect that autocrats can indeed affect popular support for the use of military force solely through their public statements on a potential conflict situation. We expect that this leader-effect can be found irrespective of opposing country characteristics, and we even expect the leader effect to be important in security-related issues as well as issues connected to cultural identity.

Hypothesis 1a: When confronted with a threat from a neighboring country, we expect citizens to be more supportive of war against that particular country if the leader in the citizen's own country expresses concerns about the situation.

Hypothesis 1b: When confronted with a threat from a neighboring country, we expect citizens to be less supportive of war against that particular country if the leader in the citizen's own country expresses lack of concerns about the situation.

Design Plan

Study type

Experiment - A researcher randomly assigns treatments to study subjects, this includes field or lab experiments. This is also known as an intervention experiment and includes randomized controlled trials.

Blinding

For studies that involve human subjects, they will not know the treatment group to which they have been assigned.

Personnel who interact directly with the study subjects (either human or non-human subjects) will not be aware of the assigned treatments. (Commonly known as "double blind")

Is there any additional blinding in this study?

No response

Contributors
Anonymous contributors

Description
This study examines whether autocratic leaders' statements about a conflict-situation with a neighboring country affects popular support for war. That is, we analyze the degree to which, and the conditions under which, authoritarian leaders can drum up support for war.

Registration type
OSF Preregistration

Date registered
February 10, 2021

Date created
February 10, 2021

Associated project
osf.io/njrcv

Category
Project

Registration DOI
10.17605/OSF.IO/BMF4W

Publication DOI
No publication DOI

Subjects
Social and Behavioral Sciences
International Relations

Study Information

Hypotheses

Authoritarian regimes are characterized by a low degree of political pluralism and a high degree of control over information. In theory, this makes it easier for authoritarian governments to drum up public support for the use of military force. We therefore expect that autocrats can indeed affect popular support for the use of military force solely through their public statements on a potential conflict situation. We expect that this leader-effect can be found irrespective of opposing country characteristics, and we even expect the leader effect to be important in security-related issues as well as issues connected to cultural identity.

Hypothesis 1a: When confronted with a threat from a neighboring country, we expect citizens to be more supportive of war against that particular country if the leader in the citizen's own country expresses concerns about the situation.

Hypothesis 1b: When confronted with a threat from a neighboring country, we expect citizens to be less supportive of war against that particular country if the leader in the citizen's own country expresses lack of concerns about the situation.

Design Plan

Study type

Experiment - A researcher randomly assigns treatments to study subjects, this includes field or lab experiments. This is also known as an intervention experiment and includes randomized controlled trials.

Blinding

For studies that involve human subjects, they will not know the treatment group to which they have been assigned.

Personnel who interact directly with the study subjects (either human or non-human subjects) will not be aware of the assigned treatments. (Commonly known as "double blind")

Study design

The argument is tested in a pre-registered survey experiment on a representative sample of around 4000 respondents in Russia. The experiment randomly exposes respondents to a fictional event—presented in a short vignette, in which a particular neighboring country (Latvia or Georgia) provokes Russia either culturally (by making it illegal for Russian children in the neighboring country to learn the Russian language in primary schools and referring to Russians as 'uncultured') or militarily (deploying long-range ballistic missiles in the neighboring country

to an area in close proximity to the Russian border and referring to Russians as ‘weak’). The descriptions of the provocation is followed by either a) no reaction from Vladimir Putin; b) an escalating statement by Vladimir Putin; or c) a deescalating statement by Vladimir Putin. After reading the vignette, respondents answer whether they support using the Russian military force against the neighboring country to stop the provocation.

Full questionnaire uploaded here (can be downloaded on pre-registration webpage)

Randomization
Treatment vignettes are completely randomized. That is, it is 100% randomized which respondents get which treatment vignette. This is administered by YouGov.

Sampling Plan
Existing Data
Registration prior to creation of data

Data collection procedures
All 4000 survey responses will be collected through YouGov. Respondents are selected through YouGov’s online panel, and the sample is representative on gender, age, region, and education.

Pilot responses on 500 respondents have been collected on November 27 2020, that is, before this pre-registration. The purpose of the pilot study is to see whether respondents fully understand the question wordings, whether randomization works as intended, and to see whether YouGov delivers the variables in the correct format. The main study with 4000 respondents will be undertaken approximately a week after this registration – that is, around end-February 2021. After the pilot study, we have decided to remove two vignette-versions which only included a provocation without any subsequent insults (Russians as ‘uncultured’ or ‘weak’), as these were not directly relevant to our main hypotheses. No other noteworthy changes were made between the pilot and the main study.

Below, we discuss vignette formulations and measurements based on the English text. However, in our experiment, respondents read the text in Russian. We intend to attach both the English version and Russian version in an Online Appendix when submitting our manuscript.

Sample size
The full sample size will be around 4000 respondents.

Respondents who fail to answer correctly on either of the two attention questions: “Please select answer number ‘2’” and “Please select answer number ‘4’”, will be removed from the study.

Also, respondents who have spent less than 120 seconds (2 minutes) on the survey will be removed.

Sample size rationale
4000 was the maximum sample size within the financial limit of this project.

Stopping rule
Only YouGov controls start and end of the data collection. Data collection is set to stop when 4000 respondents have answered the survey.

Variables
Measured variables
Main treatment variable
The main treatment variable is Vladimir Putin's reaction to the provocation—a variable we term "escalating rhetoric."

The variable is coded 0, if the vignette does not include a statement by Vladimir Putin.

The variable is coded 1, if Putin deescalates the situation. That is if the vignette states "President Putin has made the following statement regarding the situation: I am not concerned with the developments in [Country]. These actions do not constitute a direct threat to our special Russian culture or the uniqueness of the Russian people" (if cultural provocation) or "President Putin has made the following statement regarding the situation: I am not greatly concerned with the developments in [Country]. These actions do not constitute a direct threat to the security of our country or that of the Russian people" (if military provocation).

The variable is coded 2, if Putin escalates the situation. That is if the vignette states "President Putin has made the following statement regarding the situation: I look with great concern to the developments in [Country]. These actions constitute a direct threat to our special Russian culture and the uniqueness of the Russian people. They must be countered" (if cultural provocation) or "President Putin has made the following statement regarding the situation: I look with great concern to the developments in [Country]. These actions constitute a direct threat to the security of our country and the Russian people. They must be countered" (if military provocation).

In the estimation, this variable is recoded into three dummy variables. That is, a binary control condition variable (0, 1), a binary de-escalation variable (0, 1), and a binary escalation variable (0, 1). It will make no difference for the estimation whether the combined categorical variable or the dummy-specifications are used.

Main outcome variable

After reading the vignette, respondents assess whether they support military confrontation with the particular neighboring country ("Based on these developments, would you favor or oppose using Russian military force against [Country] to stop this?"). It is measured as a 5-point answer category (Favor strongly; Favor somewhat; Neither favor nor oppose; Oppose somewhat; Oppose strongly). We use this variable as a continuous 5-point variable as well as a binary dummy variable that distinguish between oppose (0) or favor (1) military confrontation.

*** Test of mechanisms ***
To examine the theoretical argument, we ask respondents, after reading the vignette, to answer a range of questions, which all tap into different theoretical logics discussed in the paper. In particular, we seek to examine whether respondents support for war is driven by feeling a) scared; b) angry; c) humiliated; d) worried for the well-being of Russians in Russia; e) worried for the well-being of Russians in the neighboring country; f) worried that the provocation threatens Russian identity; and g) worried that the provocation threatens Russian national security. We also ask whether the respondent thinks that Russia would win a potential war against the neighboring country. We intend to use these measures in a causal mediation analysis approach that examines whether and how much the main effect is mediated through these mechanisms.

Analysis Plan
Statistical models
The main estimation strategy is a series of OLS models with clustered standard errors (on respondents).

Inference criteria
Results are only considered significant at the 95% confidence level (p-value <0.05) using two-tailed tests.

Data exclusion
Respondents will be removed from the study if they fail to answer correctly on either of the two attention questions: "Please select answer number '2'" and "Please select answer number '4'". Likewise, respondents who have spent less than 120 seconds (2 minutes) on the survey will be removed.

Missing data
All respondents (except inattentive ones; see above) are included in the analysis – even if they have not answered all questions in the survey.

Exploratory analysis
When receiving our data, we are likely to explore three further patterns in the data, as we intend to scrutinize how the leader-effect might differ across different threat scenarios. First, are the

leader-effects similar across military-based and identity-based provocations? Second, are the effects similar across the two neighboring countries, Georgia and Latvia? Third, is the leader effect different across supporters and non-supporters of Vladimir Putin?

B: Survey questionnaire

Below the entire questionnaire for the survey fielded in Russia is presented.

YouGov background questions:

- Age_computed (sample)
- Gender (sample)
- Districts: (region_federal_districts (sample))
- Region: (PDL: region)
- Education level: (PDL: education_level_RU_2019)
- Personal income, gross: (PDL: profile_gross_personal_RU)
- Household income, gross: (PDL: income)

People can learn about what is going on in this country and the world from various sources. For each of the following sources, please indicate how often you use it to obtain information.

-Daily newspaper
-TV news
-Internet
-Social media

Scale:

1. Daily
2. Weekly
3. Monthly
4. Less than monthly
5. Never
6. Don't know/prefer not to answer

Which party's candidate did you vote for at the last election to the state Duma?

1. United Russia
2. The Communist Party of the Russian Federation
3. The Liberal-Democratic Party of Russia
4. Patriots of Russia
5. Yabloko
6. Progress Party / Russia of the Future
7. A Just Russia
8. Party of Growth
9. The Party of Popular Freedom PARNAS
10. Other
11. None of the above
12. Don't know
13. Refuse to answer

If elections to the State Duma were held tomorrow, which party's candidate would you likely vote for? If you don't know, which party appeals to you most?

1. United Russia
2. The Communist Party of the Russian Federation
3. The Liberal-Democratic Party of Russia
4. Yabloko
5. Progress Party / Russia of the Future
6. A Just Russia
7. Party of Growth
8. The Party of Popular Freedom PARNAS
9. Other
10. None of the above
11. Don't know
12. Refuse to answer

Recently, important political events occurred in our country and in a neighboring country. We are interested in your opinion on these events.

This survey includes 20 short questions and there are no right or wrong answers.

We reserve the right to reject anyone who simply clicks through the survey without carefully reading and answering the questions.

All answers are completely anonymous.

Thank you!

Please select answer number "2".

<1> 1
<2> 2
<3> 3
<4> 4
<5> 5

In general, do you approve or disapprove of the way Vladimir Putin is handling his job as President?

<5> Strongly approve
<4> Somewhat approve
<3> Neither approve nor disapprove
<2> Somewhat disapprove
<1> Strongly disapprove
<977> Don't know

Thank you! You will now be presented with a short description of a recent event in a neighboring country. Please read it carefully and answer the following questions.

Text – if Splitsample=1 Vignette1
Yesterday, [Country]'s government passed a law that changes the design of their country's passport to make it look less similar to the Russian ones.

Text – if Splitsample=2 Vignette2
Yesterday, [Country]'s government passed a law that makes it illegal for Russian children in [Country] to learn the Russian language in primary schools. During the following press conference, [Country]'s prime minister referred to Russians as 'uncultured'.

Text – if Splitsample=3 Vignette3
Yesterday, [Country]'s government passed a law that makes it illegal for Russian children in [Country] to learn the Russian language in primary schools. During the following press conference, [Country]'s prime minister referred to Russians as 'uncultured'.

President Putin has made the following statement regarding the situation: I look with great concern to the developments in [Country]. These actions constitute a direct threat to our special Russian culture and the uniqueness of the Russian people. They must be countered.

Text – if Splitsample=4 Vignette4
Yesterday, [Country]'s government passed a law that makes it illegal for Russian children in [Country] to learn the Russian language in primary schools. During the following press conference, [Country]'s prime minister referred to Russians as 'uncultured'.

President Putin has made the following statement regarding the situation: I am not concerned with the developments in [Country]. These actions do not constitute a direct threat to our special Russian culture or the uniqueness of the Russian people.

Text – if Splitsample=5 Vignette5
Yesterday, [Country]'s government passed a law that provides funding for the deployment of long-range, ballistic missiles to an area in close proximity to the Russian border. During the following press conference, [Country]'s prime minister referred to Russians as 'weak'.

Text – if Splitsample=6 Vignette6
Yesterday, [Country]'s government passed a law that provides funding for the deployment of long-range, ballistic missiles to an area in close proximity to the Russian border. During the following press conference, [Country]'s prime minister referred to Russians as 'weak'.

President Putin has made the following statement regarding the situation: I look with great concern to the developments in [Country]. These actions constitute a direct threat to the security of our country and the Russian people. They must be countered.

Text – if Splitsample=7 Vignette7
Yesterday, [Country]'s government passed a law that provides funding for the deployment of long-range, ballistic missiles to an area in close proximity to the Russian border. During the following press conference, [Country]'s prime minister referred to Russians as 'weak'.

President Putin has made the following statement regarding the situation: I am not greatly concerned with the developments in [Country]. These actions do not constitute a direct threat to the security of our country or that of the Russian people.

Based on these developments, would you favor or oppose using Russian military force against [Country] to stop this?

1 ○	2 ○	3 ○	4 ○	5 ○
Favor strongly	Favor somewhat	Neither favor nor oppose	Oppose somewhat	Oppose strongly

---

To what extent do you agree with the following statements?

I feel <u>scared</u> when I read about the event in [Country]

1. I agree very much
2. I somewhat agree
3. Neither/nor
4. I somewhat disagree
5. I disagree at all

I feel angry when I read about the event in [Country]

1. I agree very much
2. I somewhat agree
3. Neither/nor
4. I somewhat disagree
5. I disagree at all

I feel humiliated when I read about the event in [Country]

1. I agree very much
2. I somewhat agree
3. Neither/nor
4. I somewhat disagree
5. I disagree at all

I am worried for the well-being of Russians in Russia when I read about the event in [Country]

1. I agree very much
2. I somewhat agree
3. Neither/nor
4. I somewhat disagree
5. I disagree at all

I am worried for the well-being of Russians in [Country] when I read about the event in [Country]

1. I agree very much
2. I somewhat agree
3. Neither/nor
4. I somewhat disagree
5. I disagree at all

I am worried that the event in [Country] threatens Russian identity

1. I agree very much
2. I somewhat agree
3. Neither/nor
4. I somewhat disagree
5. I disagree at all

I am worried that the event in [Country] threatens Russian national security

1. I agree very much
2. I somewhat agree
3. Neither/nor
4. I somewhat disagree
5. I disagree at all

If a war should break out with [Country], do you think Russia will win this war?

1 ○	2 ○	3 ○	4 ○	5 ○
Yes, definitely	Yes, probably	Neither/nor	Probably not	Definitely not

---

Which of these countries did you just read about?

**1** ○	**2** ○	**3** ○	4 ○	5 ○
Denmark	Brazil	Congo	Latvia	Georgia

Please select answer number “4”.

<1> 1
<2> 2
<3> 3
<4> 4
<5> 5

Thank you for participating in this survey!

You have read about protests within Russia and about events in a neighboring country. Some of the information provided about these events may have made you feel worried.

We would like to stress that the portrayed events are not real. They are fictional and have been constructed for research purposes only. No protests occurred at Tverskaya Ulitsa in the way described, and our relationship with Latvia or Georgia has not deteriorated as portrayed in the survey.

This type of research is of great scientific importance because it allows us to study important causes of peace and conflict, which will hopefully become useful for both academic scholarship and international governance. We would therefore like to thank you again for your participation.

You can also contact the research team with any questions, concerns, or complaints that you may have about the research by e-mailing XXXXXXX.

C: Ethical considerations

Survey methodology

We examine Russian citizens' support for war in a pre-registered survey experiment, administered using YouGov, on a representative sample on gender, age, and geography of 4,144 respondents in Russia. The data was collected from February 12 to March 11, 2021. The survey data is registered at the Data Protection Unit at the authors' University. All potential ethical issues have been continuously discussed and cleared with YouGov and their Russian survey partners.

The experiment randomly exposes respondents to a fictional event presented in a detailed yet concise vignette. The leadership of a neighboring country (randomly presented as either Latvia or Georgia) performs actions that Russians may perceive as threatening and deliver insulting statements against Russians.

The structure of the vignette experiment follows a clear format. Respondents are assigned to one vignette, which can be either a control condition, a provocation from one of the neighboring countries, a provocation followed by a de-escalating statement by Russian President Putin, or a provocation followed by an escalating statement by Putin. The vignettes describe realistic scenarios and imitate typical descriptions and phrases often used by newspaper articles in Russia. In total, this leaves us with four main conditions. Respondents are assigned to one of these randomly. After reading the vignette, respondents answer whether they support using Russian military force against Georgia (or Latvia).

Recruitment

All survey answers are collected online from voluntary participants who are already part of YouGov's online survey panel (or YouGov partners' online survey panel). Potential respondents receive an e-mail with a request to participate in the survey. The e-mail includes a short description of the survey, guarantees of participant anonymity, information on data protection and storage, payment details, and a link to the survey with instructions. It is made clear to all potential respondents that participation is completely voluntary.

Consent

All participants are recruited based on informed consent; that is, potential participants are first told about the study details (see above) and then asked whether they want to participate. If they agree to participate in the survey, respondents can click on the attached link that takes them to the survey. Before answering the questions in the survey, they are once again presented with information about the survey and told that all answers are completely anonymous. Respondents can withdraw their consent to participate at any time during the survey without losing payments. Moreover, each survey question includes a "don't know"/"prefer not to answer" option, which allows respondents to participate in only some parts of the survey while declining to participate in other parts of the survey without any deductions in payments. In so doing, respondents are neither directly nor indirectly forced to participate in the full survey even after granting initial consent.

Compensation

All respondents receive fair compensation for their participation. The exact amount paid varies depending on which "survey bundle" the respondent chooses to undertake. After finishing the

survey, respondents can also choose to receive YouGov points that can be converted into vouchers at the YouGov online store (or YouGov partner stores). No matter what option participants choose, the payment they receive is indeed generous; converted into monetary terms, it exceeds the hourly minimum wage in Russia. Respondents are told about the size of the payment before deciding whether or not to participate in the survey to ensure that all participants make an informed decision.

Data protection and confidentiality

All survey responses are 100% anonymous, and the experiment and the data collection process have been approved and registered at the Data Protection Unit at the university of the authors. By assigning our project to this institutional review board, our survey data collection has been subject to an array of restrictions and guidelines monitored by the board. For example, the dataset with survey responses includes only a randomized ID along with all survey answers. Therefore, no answers can be traced back to individual respondents. In addition, YouGov has provided a signed document guaranteeing that all data management on their behalf complies with national legal regulations and ethical standards. Moreover, the Institutional review board continuously monitors our handling of the data and provides guidelines for how to make data public while at the same time ensuring data protection and confidentiality.

We see this as our main ethical issue. In today's Russia, citizens risk severe punishment for providing statements the regime considers disloyal, especially regarding Russian war efforts in neighboring countries. Therefore, ensuring the anonymity of respondents and proper storage of sensitive survey data is vital.

Deception, misrepresentation, and potential harm

Although respondents received fictional information in the survey vignettes, they were not deceived. That is, the survey includes no identity deception (i.e., deception about who is undertaking the survey), no activity deception (i.e., deception about the purpose of the survey), and no motivation deception (i.e., deception about the reasons for the research or how data will be used).

It does include misinformation, however, as the vignettes describe fictional events without initially mentioning that they are fictional. Such randomized treatment manipulation is pivotal in estimating the causal effect of provocations from neighboring countries on citizens' willingness to support war. Still, it comes with the risk that the Russian respondents may lose trust in mainstream news media outlets and academic research. In addition, by reading about provocations from neighboring countries, there is a risk of Russian citizens generally becoming more supportive of violence and the use of military force against the countries they read about in the survey.

We took several measures to mitigate such ethical risks. Most importantly, we constructed vignettes that are fictional but still very much resemble stories, phrases, and words that have appeared in the regime-controlled Russian media. In other words, the vignette descriptions are very similar to the kind of propaganda stories to which Russians are frequently exposed—and particularly so in the months leading up to and during the 2022 war in Ukraine. This means that we exposed respondents to information that they would likely encounter or already have encountered anyway. Moreover, we deliberately used relatively vague event descriptions and mildly formulated Putin statements to further minimize the risk that respondents' opinions

concerning particular neighboring countries are affected in the long run by participation in our survey.

Still, to ensure that respondents are, in fact, not deceived and understand that the vignettes are fictitious, respondents received thorough debriefing information at the end of the survey. We formulated the debrief in an easy-to-understand manner by relying on everyday Russian formulations. Though we do not test for it, the brief debrief (which quickly points out that the event informations provided in the survey are fictious) hopefully ensured that all respondents understand that the vignette information they received is not real. We explicitly stated that Russian relations with Latvia/Georgia had not deteriorated as described in the survey, and we explained that the use of such fictitious events is important for research on and our understanding of the causes of peace and conflict.

"Thank you for participating in this survey!

You have read about protests within Russia and about events in a neighboring country. Some of the information provided about these events may have made you feel worried.

We would like to stress that the portrayed events are not real. They are fictional and have been constructed for research purposes only. No protests occurred at Tverskaya Ulitsa in the way described, and our relationship with Latvia or Georgia has not deteriorated as portrayed in the survey.

This type of research is of great scientific importance because it allows us to study important causes of peace and conflict, which will hopefully become useful for both academic scholarship and international governance. We would therefore like to thank you again for your participation.

You can also contact the research team with any questions, concerns, or complaints that you may have about the research by e-mailing xxxxxx."

In sum, taking these measures, we explicitly tried to reduce the risk of any harm as a result of the fictional vignettes. Moreover, by providing an e-mail address, all respondents have the opportunity to write to the research team with any possible complaints. So far, no one has contacted us or filed a complaint with YouGov or their Russian survey partner.

<u>The expected societal benefit of the research</u>

Despite the ethical challenges discussed above, we believe that the potential societal benefits of this research are too important to neglect. Notably, the findings from our survey experiment add to our understanding of one of the worst geopolitical catastrophes in Europe since the end of the Cold War. The Russian war in Ukraine has already led to tens, maybe even hundreds, of thousands of military and civilian casualties, millions of refugees and internally displaced, vast destruction of Ukrainian cities and infrastructure, and not least deep animosity between Russia and the West and an insecure and vulnerable European security context. The key to stopping this devastating war is a better understanding of what drives it. Our survey analysis provides solid insights into how and how much the Russian regime could shape public support for the military invasion through propaganda and leader rhetoric around the time of the invasion. It also

demonstrates some of the factors that are important for whether Russians withdraw support for the military campaign in Ukraine. Our findings can thus help policymakers and analysts better to understand the conflict and, hopefully, improve the policy response of the West.

D: Distribution of Putin approval
Before reading the vignette, respondents declared their approval/disapproval of President Vladimir Putin.

> *In general, do you approve or disapprove of the way Vladimir Putin is handling his job as President?*

The answer categories are measured on a five-point scale (strongly disapprove, somewhat disapprove, neither approve nor disapprove, somewhat approve, strongly approve). Asking respondents about the president's performance in a repressive autocracy as Russia can induce social desirability bias ¬¬¬– that is, there is a potential risk that respondents will not declare their true opinions due to fear of repercussions. Yet, as shown from the distribution of answers in Fig. S1 below, respondents are equally likely to report their disapproval of the president as they are to express their approval. This suggests that respondents trust and respond well to our repeated reassurances of anonymity throughout the survey.

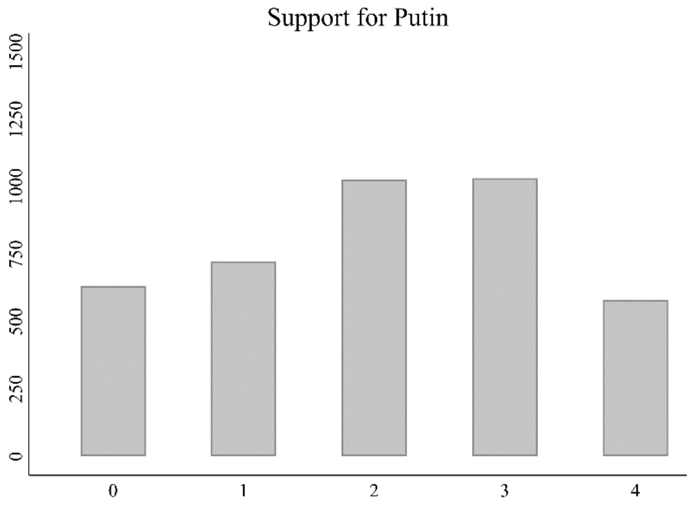

Fig. S1. Distribution of Putin approval. Distribution of support for President Putin. 0 = Strongly disapprove; 1 = Somewhat disapprove; 2 = Neither approve nor disapprove; 3 = Somewhat approve; 4 = Strongly approve

E: Regression table of main results

	Figure 1 (left panel) (1)	Figure 1 (right panel) (2)	Figure 2 (left panel) (3)	Figure 2 (right panel) (4)
Prov	0.415*	0.458*	-0.038	0.867*
	(0.057)	(0.100)	(0.114)	(0.115)
Prov+De	0.445*	0.472*	-0.030	0.962*
	(0.056)	(0.098)	(0.106)	(0.115)
Prov+Esc	0.662*	0.581*	0.139	1.007*
	(0.057)	(0.102)	(0.113)	(0.125)
Support Putin		0.395*	0.400*	0.390*
		(0.104)	(0.104)	(0.106)
Prov x Support Putin		-0.004	-0.067	0.262
		(0.138)	(0.153)	(0.161)
Prov+De x Support Putin		0.041	0.140	-0.031
		(0.134)	(0.150)	(0.154)
Prov+Esc x Support Putin		0.265	0.189	0.356*
		(0.140)	(0.160)	(0.164)
Gender		0.143*	0.133*	0.165*
		(0.047)	(0.057)	(0.058)
Age				
25-34		-0.194*	-0.308*	-0.097
		(0.087)	(0.118)	(0.108)
35-44		-0.136	-0.349*	0.050
		(0.088)	(0.117)	(0.110)
45-54		-0.328*	-0.561*	-0.133
		(0.092)	(0.121)	(0.114)
55+		-0.538*	-0.830*	-0.296*
		(0.086)	(0.113)	(0.108)
Region				
Northwestern Federal District		-0.076	-0.097	0.060
		(0.092)	(0.107)	(0.113)
Southern Federal District		-0.156	-0.181	-0.149
		(0.081)	(0.098)	(0.100)
North Caucasus Federal District		0.049	0.113	-0.044
		(0.115)	(0.147)	(0.144)
Volga Federal District		-0.001	-0.034	0.019
		(0.066)	(0.079)	(0.082)
Ural federal district		-0.036	-0.112	-0.012
		(0.092)	(0.110)	(0.114)
Siberian Federal District		-0.086	-0.039	-0.193*
		(0.078)	(0.095)	(0.098)
Far Eastern Federal District		0.064	-0.108	0.272
		(0.140)	(0.161)	(0.154)
Income				
100,001 – 200,000 RUB		-0.276*	-0.130	-0.349*
		(0.103)	(0.132)	(0.129)
200,001 – 300,000 RUB		-0.038	-0.065	0.008
		(0.109)	(0.138)	(0.128)
300,001 – 400,000 RUB		-0.069	-0.039	-0.140

		(0.108)	(0.139)	(0.131)
400,001 – 500,000 RUB		-0.188<br>(0.101)	-0.191<br>(0.125)	-0.154<br>(0.123)
500,001 – 1,000,000 RUB		-0.218*<br>(0.089)	-0.259*<br>(0.113)	-0.171<br>(0.108)
More than 1,000,000 RUB		-0.319*<br>(0.096)	-0.356*<br>(0.120)	-0.271*<br>(0.116)
Difficult to say		-0.133<br>(0.142)	-0.271<br>(0.190)	-0.200<br>(0.160)
Constant	1.212*<br>(0.043)	1.220*<br>(0.146)	1.462*<br>(0.175)	0.993*<br>(0.174)
Observations	4144	2969	1694	1703

Table S1. Regression table of main analyses in the article. Robust standard errors in parentheses * $p < 0.05$

F: Main results for Latvia and Georgia separately

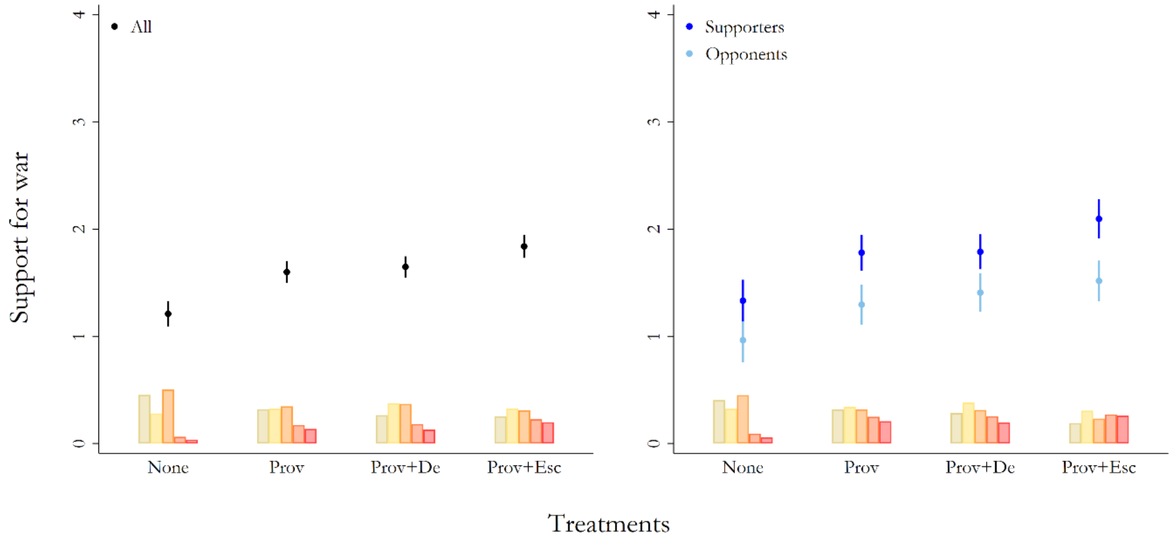

Fig. S2. Figure 1 (Latvia). The X-axis presents the control condition and the three treatment conditions. The Y-axis denotes the estimated values of support for war (0-4) with 95% confidence intervals. The left panel presents the main results of the full sample given by Model 1. The right panel illustrates the main results across Putin supporters (dark blue) and opponents (light blue) given by Model 2. The bars at the bottom of each graph illustrate the distribution in respondents' answers on support for war across each treatment condition (Green=Oppose strongly; Yellow=Oppose somewhat; Orange=Neither favor nor oppose; Dark orange=Favor somewhat; Red=Favor strongly). In the right panel, the bars only show distributions among Putin supporters. Only treatments with Latvia are used for these analyses.

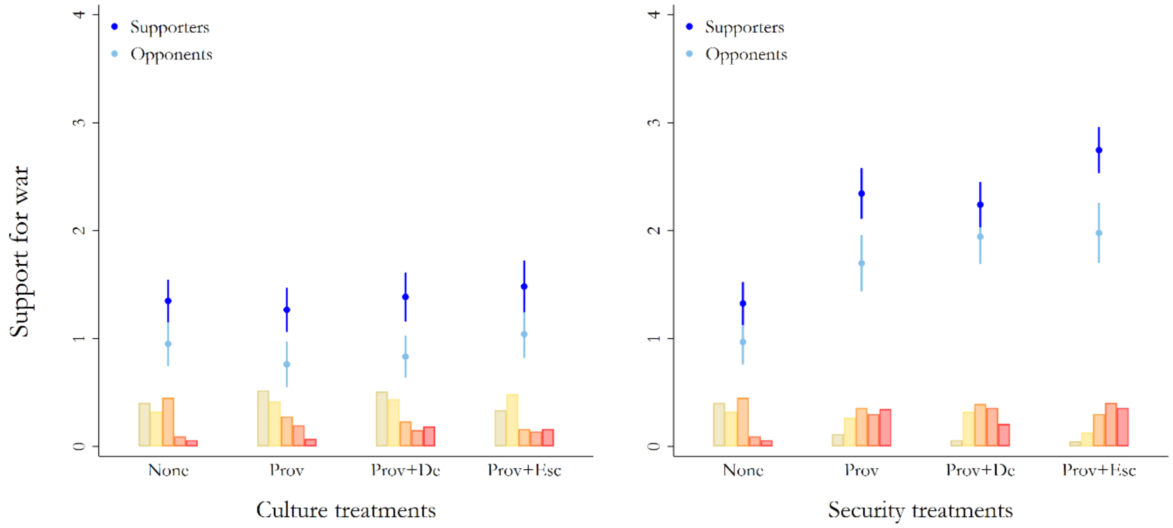

Fig. S3. Figure 2 (Latvia). The X-axis presents the control condition and the three treatment conditions. The Y-axis denotes the estimated values of support for war (0-4) with 95% confidence intervals. Results are presented across supporters (dark blue) and opponents (light blue) of President Putin given by Model 2. The left panel presents results for culture treatments only. The right panel presents results for security treatments only. The bars at the bottom of each graph illustrate the distribution in respondents' answers on support for war across each treatment condition (Green=Oppose strongly; Yellow=Oppose somewhat; Orange=Neither favor nor oppose; Dark orange=Favor somewhat; Red=Favor strongly) among Putin supporters. Only treatments with Latvia are used for these analyses.

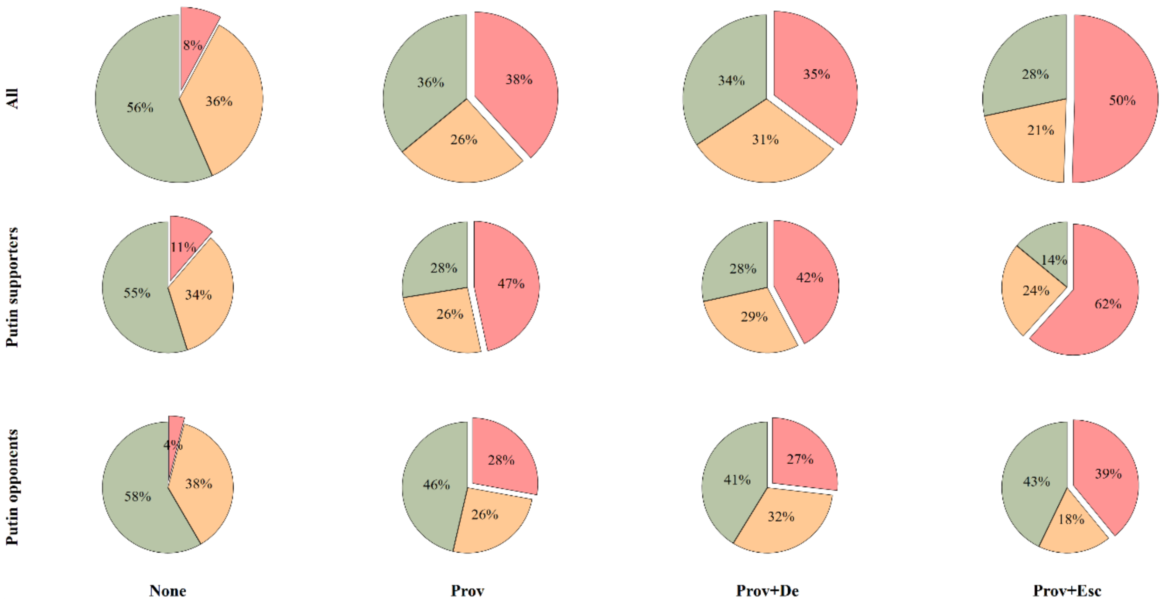

Fig. S4. Figure 3 (Latvia). Charts of the estimated distributions of support for war across security treatment conditions. Green equals opposition to war (oppose strongly or oppose somewhat); Orange is undecided/indifferent (neither favor nor oppose); and red equals support for war (favor somewhat or favor strongly). The top row shows distributions across all respondents, the middle row across Putin supporters, and the bottom row across Putin opponents. Only treatments with Latvia are used for these analyses.

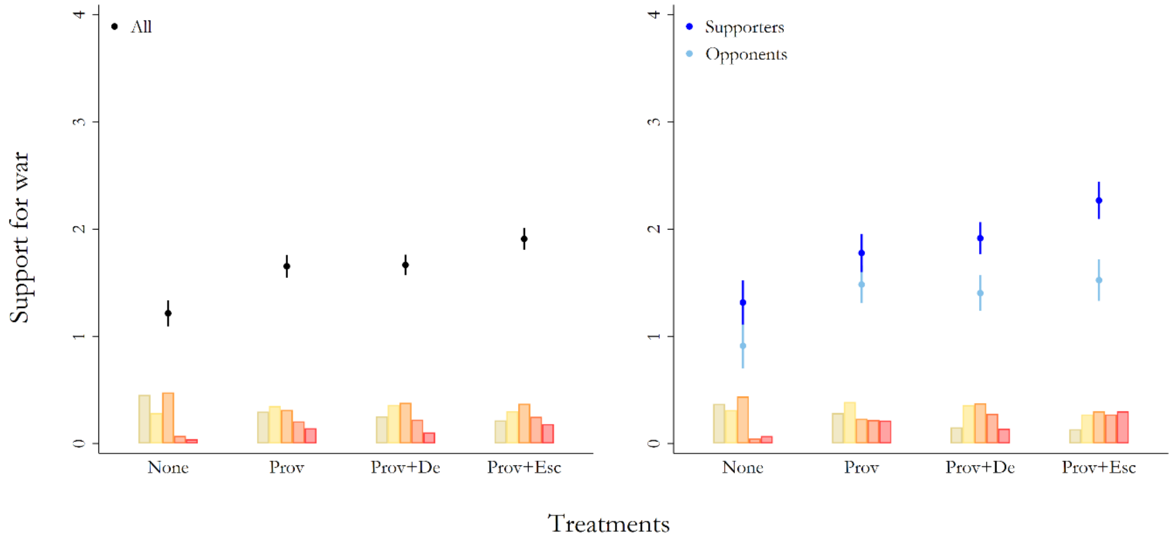

Fig. S5. Figure 1 (Georgia). The X-axis presents the control condition and the three treatment conditions. The Y-axis denotes the estimated values of support for war (0-4) with 95% confidence intervals. The left panel presents the main results of the full sample given by Model 1. The right panel illustrates the main results across Putin supporters (dark blue) and opponents (light blue) given by Model 2. The bars at the bottom of each graph illustrate the distribution in respondents' answers on support for war across each treatment condition (Green=Oppose strongly; Yellow=Oppose somewhat; Orange=Neither favor nor oppose; Dark orange=Favor somewhat; Red=Favor strongly). In the right panel, the bars only show distributions among Putin supporters. Only treatments with Georgia are used for these analyses.

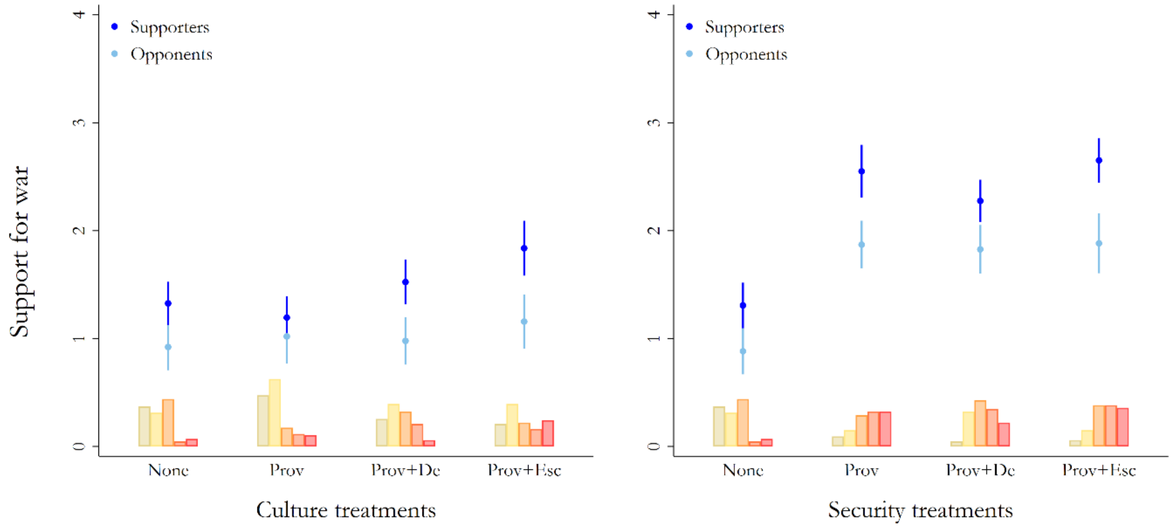

Fig. S6. Figure 2 (Georgia). The X-axis presents the control condition and the three treatment conditions. The Y-axis denotes the estimated values of support for war (0-4) with 95% confidence intervals. Results are presented across supporters (dark blue) and opponents (light blue) of President Putin given by Model 2. The left panel presents results for culture treatments only. The right panel presents results for security treatments only. The bars at the bottom of each graph illustrate the distribution in respondents' answers on support for war across each treatment condition (Green=Oppose strongly; Yellow=Oppose somewhat; Orange=Neither favor nor oppose; Dark orange=Favor somewhat; Red=Favor strongly) among Putin supporters. Only treatments with Georgia are used for these analyses.

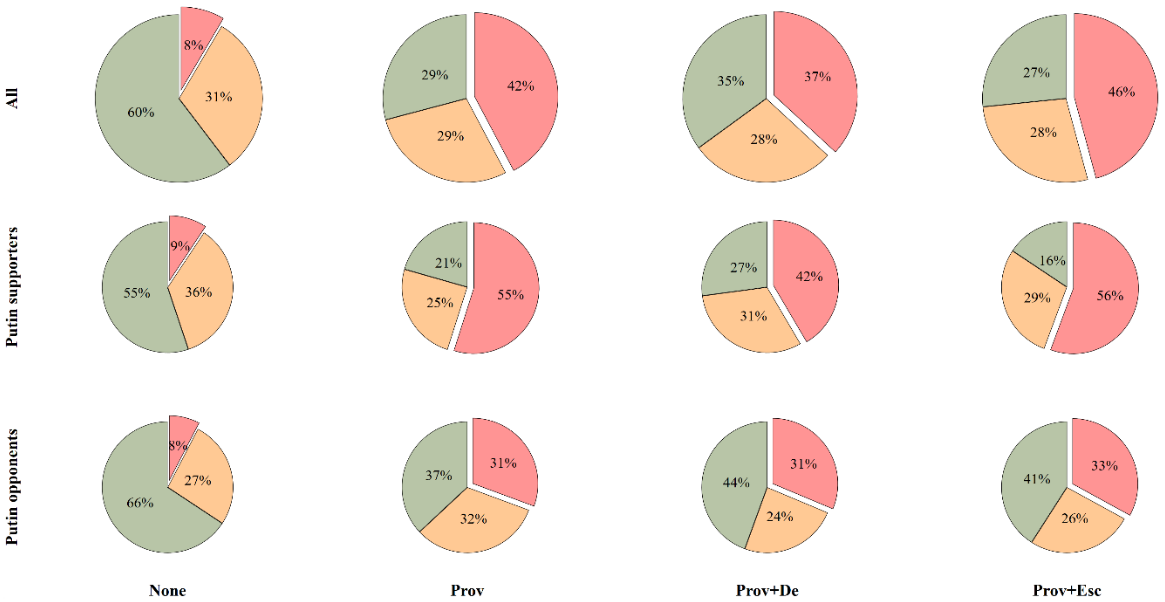

Fig. S7. Figure 3 (Georgia). Charts of the estimated distributions of support for war across security treatment conditions. Green equals opposition to war (oppose strongly or oppose somewhat); Orange is undecided/indifferent (neither favor nor oppose); and red equals support for war (favor somewhat or favor strongly). The top row shows distributions across all respondents, the middle row across Putin supporters, and the bottom row across Putin opponents. Only treatments with Georgia are used for these analyses.

G: Assessing the representativeness of the survey-sample

	Variable	Sample	Population
**Gender**	Female	51,93	53.51
	Male	48,07	46.49
**Age**	18-24	8.21	9.08
	25-34	22.55	16.86
	35-44	22.73	20.67
	45-54	18.89	16.43
	55+	27.63	36.97
**Region**	Central Federal District	29.99	27.40
	Northwestern Federal District	9.21	9.46
	Southern Federal District	10.63	11.38
	North Caucasus Federal District	4.96	6.91
	Volga Federal District	21.61	19.66
	Ural Federal District	8.26	8.36
	Siberian Federal District	11.92	11.41
	Far Eastern Federal District	3.42	5.42

Table S2. Representativeness of survey-sample versus population census data. The table compares sample statistics with official Russian census data from the Federal State Statistics Service for 2021 on gender, age, and region.

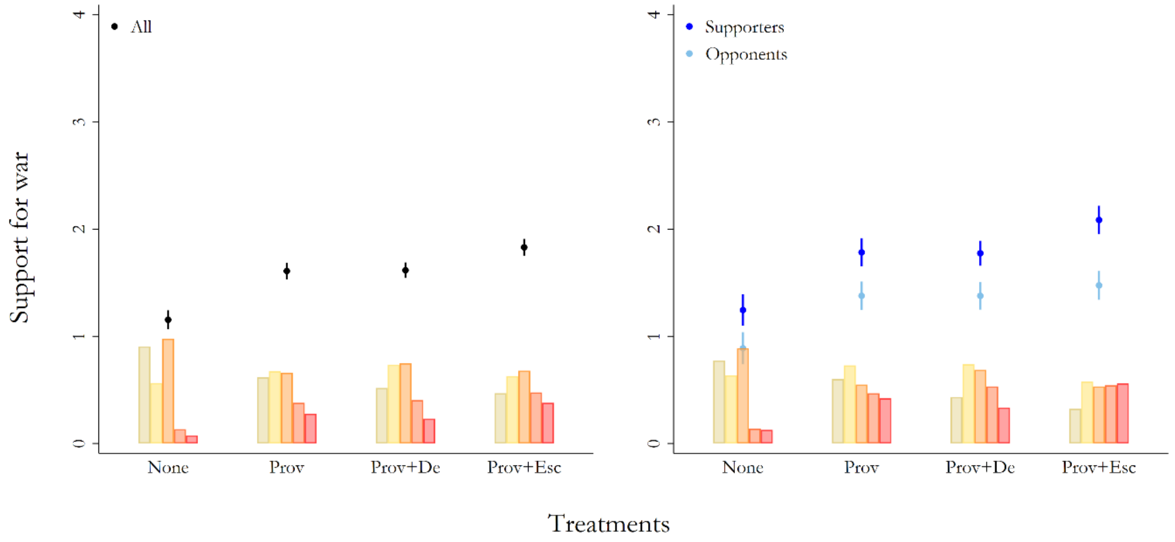

Fig. S8. Figure 1 with weights. The X-axis presents the control condition and the three treatment conditions. The Y-axis denotes the estimated values of support for war (0-4) with 95% confidence intervals. The left panel presents the main results of the full sample given by Model 1. The right panel illustrates the main results across Putin supporters (dark blue) and opponents (light blue) given by Model 2. The bars at the bottom of each graph illustrate the distribution in respondents' answers on support for war across each treatment condition (Green=Oppose strongly; Yellow=Oppose somewhat; Orange=Neither favor nor oppose; Dark orange=Favor somewhat; Red=Favor strongly). In the right panel, the bars only show distributions among Putin supporters. All analyses use survey weights.

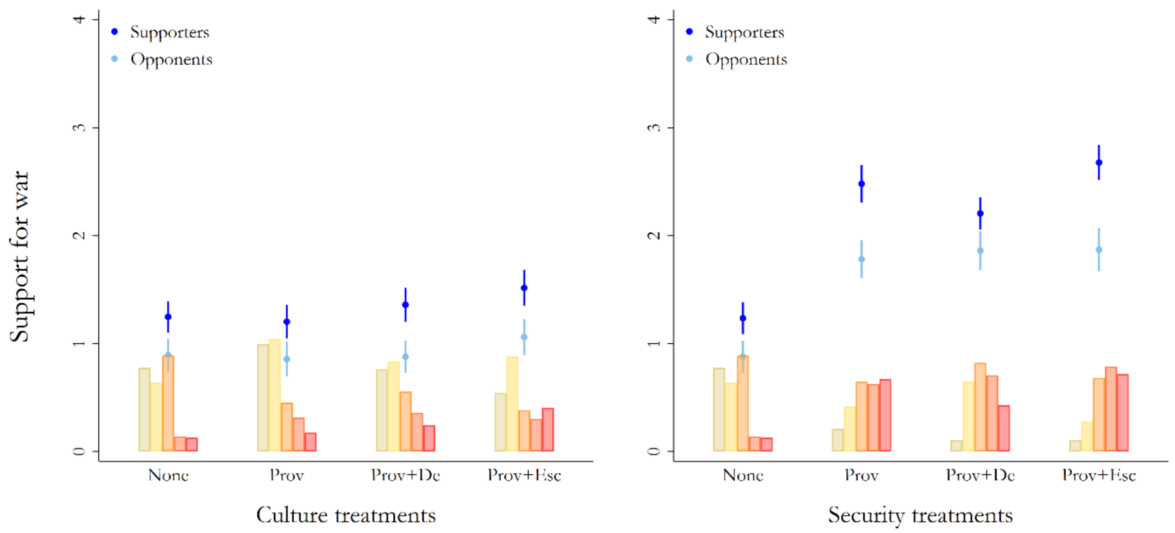

Fig. S9. Figure 2 with weights. The X-axis presents the control condition and the three treatment conditions. The Y-axis denotes the estimated values of support for war (0-4) with 95% confidence intervals. Results are presented across supporters (light blue) and opponents (dark blue) of President Putin given by Model 2. The left panel presents results for culture treatments only. The right panel presents results for security treatments only. The bars at the bottom of each graph illustrate the distribution in respondents' answers on support for war across each treatment condition (Green=Oppose strongly; Yellow=Oppose somewhat; Orange=Neither favor nor oppose; Dark orange=Favor somewhat; Red=Favor strongly) among Putin supporters. All analyses use survey weights.

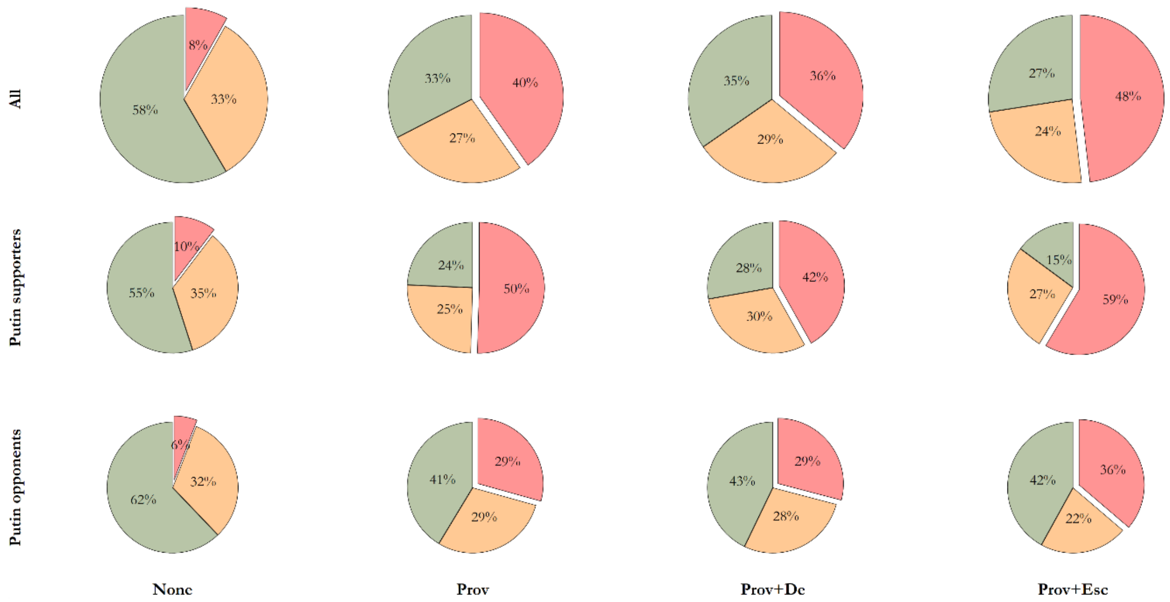

Fig. S10. Figure 3 with weights. Charts of the estimated distributions of support for war across security treatment conditions. Green equals opposition to war (oppose strongly or oppose somewhat); Orange is undecided/indifferent (neither favor nor oppose); and red equals support for war (favor somewhat or favor strongly). The top row shows distributions across all respondents, the middle row across Putin supporters, and the bottom row across Putin opponents. All analyses use survey weights.

H: Scrutinizing the effect of culture treatments

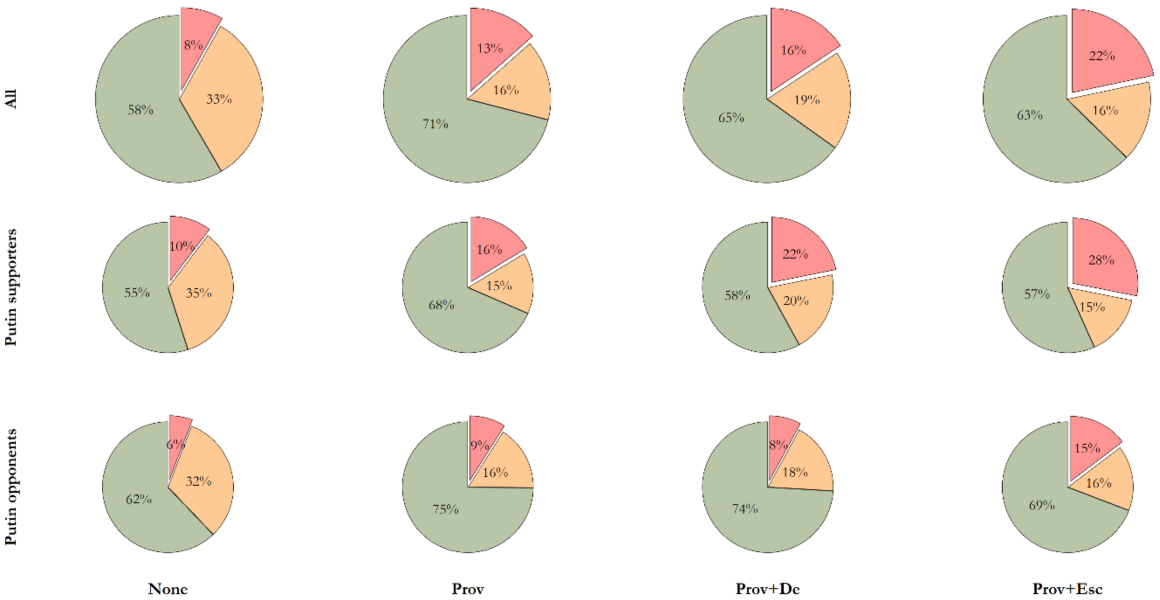

Fig. S11. Support for war across culture treatments. Charts of the estimated distributions of support for war across security treatment conditions. Green equals opposition to war (oppose strongly or oppose somewhat); Orange is undecided/indifferent (neither favor nor oppose); and red equals support for war (favor somewhat or favor strongly). The top row shows distributions across all respondents, the middle row across Putin supporters, and the bottom row across Putin opponents.

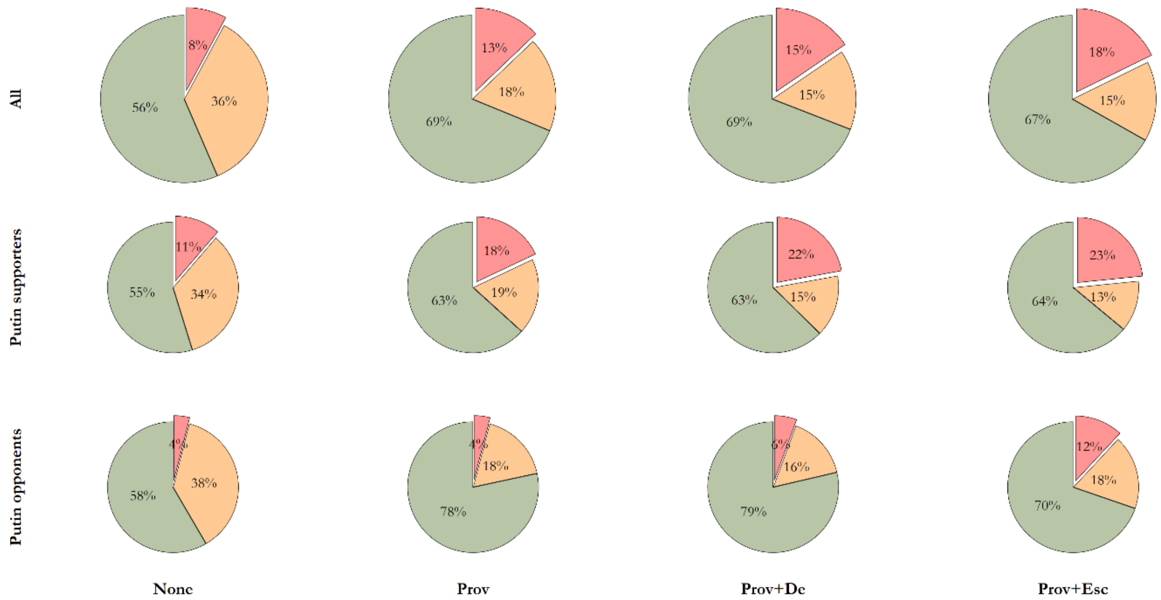

Fig. S12. Support for war across culture treatments (Latvia). Charts of the estimated distributions of support for war across security treatment conditions. Green equals opposition to war (oppose strongly or oppose somewhat); Orange is undecided/indifferent (neither favor nor oppose); and red equals support for war (favor somewhat or favor strongly). The top row shows distributions across all respondents, the middle row across Putin supporters, and the bottom row across Putin opponents.

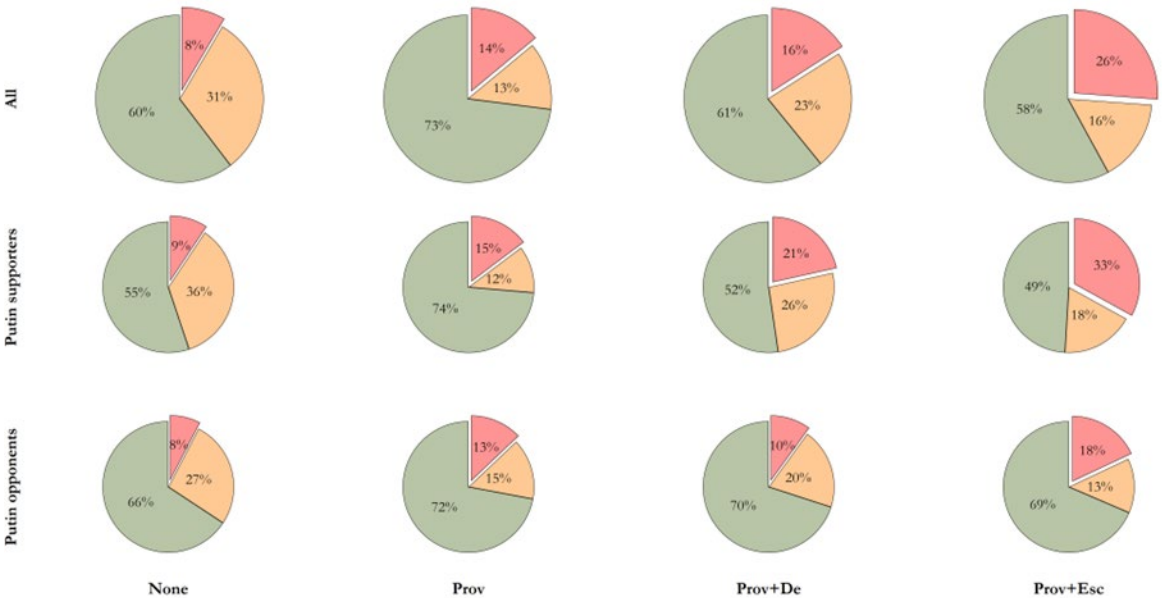

Fig. S13. Support for war across culture treatments (Georgia). Charts of the estimated distributions of support for war across security treatment conditions. Green equals opposition to war (oppose strongly or oppose somewhat); Orange is undecided/indifferent (neither favor nor oppose); and red equals support for war (favor somewhat or favor strongly). The top row shows distributions across all respondents, the middle row across Putin supporters, and the bottom row across Putin opponents.

I: Examining the impact of media usage

	Control for media usage (1)	Newspaper daily only (2)	Television daily only (3)	Internet daily only (4)	Social media daily only (5)
Prov	0.422*	0.156	0.464*	0.407*	0.310*
	(0.058)	(0.220)	(0.072)	(0.060)	(0.068)
Prov+De	0.438*	0.412	0.506*	0.451*	0.400*
	(0.057)	(0.213)	(0.069)	(0.058)	(0.067)
Prov+Esc	0.676*	0.414	0.748*	0.650*	0.593*
	(0.059)	(0.231)	(0.073)	(0.060)	(0.069)
Newspaper daily	0.397*				
	(0.074)				
Television daily	0.137*				
	(0.042)				
Internet daily	-0.230*				
	(0.073)				
Social Media daily	0.098*				
	(0.048)				
Constant	1.218*	1.745*	1.204*	1.209*	1.296*
	(0.080)	(0.178)	(0.053)	(0.045)	(0.053)
Observations	3881	376	2632	3784	2986

Table S3. Regression table of analyses with controls and split-samples of media usage. Robust standard errors in parentheses * $p < 0.05$.

J: Results from the pilot study

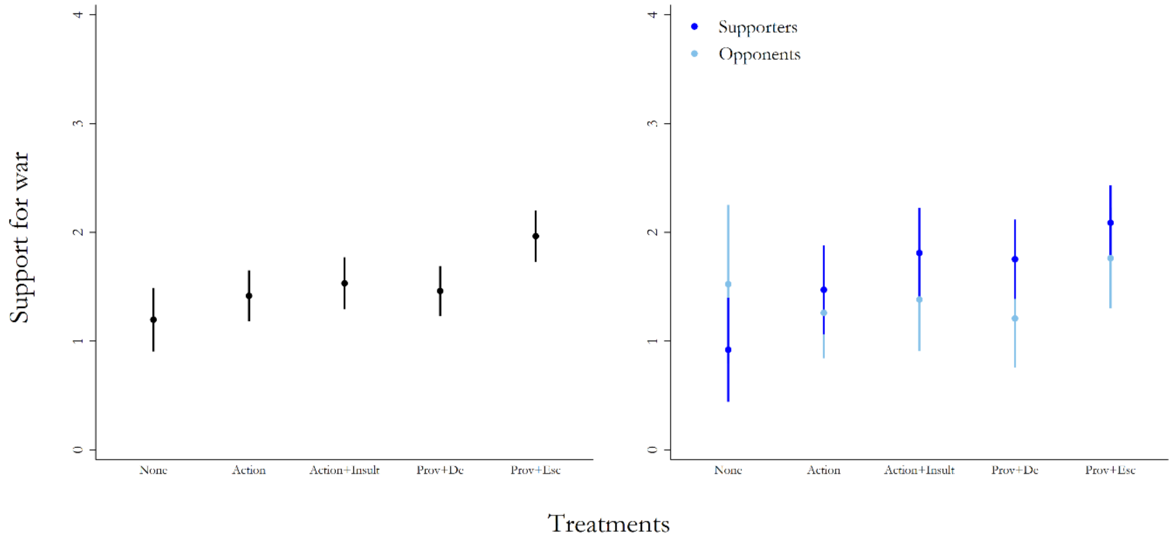

Fig. S14. Pilot results. The X-axis presents the control condition and the three treatment conditions. The Y-axis denotes the estimated values of support for war (0-4) with 95% confidence intervals. The left panel presents the main results of the full sample given by Model 1. The right panel illustrates the main results across Putin supporters (dark blue) and opponents (light blue) given by Model 2. The bars at the bottom of each graph illustrate the distribution in respondents' answers on support for war across each treatment condition (Green=Oppose strongly; Yellow=Oppose somewhat; Orange=Neither favor nor oppose; Dark orange=Favor somewhat; Red=Favor strongly). In the right panel, the bars only show distributions among Putin supporters.

Data S1. Data files for analyses. Contains:

data_1
Deplyment_of_missiles
Russian_Security
Russianspeakers_security
Russophobia
Threat_to_Russia
Worry_Concern

Data and dofile can be downloaded at:
https://dataverse.harvard.edu/dataset.xhtml?persistentId=doi:10.7910/DVN/KE7XVX
